# Glycol bearing perylene monoimide based non-fullerene acceptors with increased dielectric permittivity

**DOI:** 10.1007/s00706-022-02956-2

**Published:** 2022-07-29

**Authors:** Peter Fürk, Jakob Hofinger, Matiss Reinfelds, Thomas Rath, Heinz Amenitsch, Markus Clark Scharber, Gregor Trimmel

**Affiliations:** 1 Institute for Chemistry and Technology of Materials (ICTM), NAWI Graz, Graz University of Technology, Stremayrgasse 9, 8010 Graz, Austria; 2 Linz Institute for Organic Solar Cells (LIOS), Institute of Physical Chemistry, Johannes Kepler University Linz, Altenbergerstrasse 69, 4040 Linz, Austria; 3 Institute of Inorganic Chemistry, NAWI Graz, Graz University of Technology, Stremayrgasse 9, 8010 Graz, Austria

**Keywords:** Organic photovoltaics, Material science, Organic semiconductors, Density functional theory, Optical properties

## Abstract

Perylene monoimide based electron acceptors have great properties for use in organic solar cells, like thermal stability, strong absorption, and simple synthesis. However, they typically exhibit low values for the dielectric permittivity. This hinders efficient exciton dissociation, limiting the achievable power conversion efficiencies. In this work, we present the synthesis and utilization of two new acceptor–donor-acceptor (A-D-A) molecules, comprising perylene monoimide as electron withdrawing A unit. Oligo ethylene glycol side chain modified carbazole (PMI-[**C-OEG**]) and fluorene (PMI-[**F-OEG**]) linkers were used as electron rich D units, respectively. The polar side chains are expected to increase the polarizability of the molecules and, thus, their permittivity according to the Clausius–Mossotti relationship. We found that the incorporation of glycol chains improved the dielectric properties of both materials in comparison to the reference compounds with alkyl chains. The permittivity increased by 18% from 3.17 to 3.75 for the carbazole-based non-fullerene acceptor PMI-[**C-OEG**] and by 12% from 3.10 to 3.47 for the fluorene-based acceptor PMI-[**F-OEG**]. The fabricated solar cells revealed power conversion efficiencies of 3.71 ± 0.20% (record 3.92%) with PMI-[**C-OEG**], and 1.21 ± 0.06% (record 1.51%) with PMI-[**F-OEG**].

**Graphical abstract:**

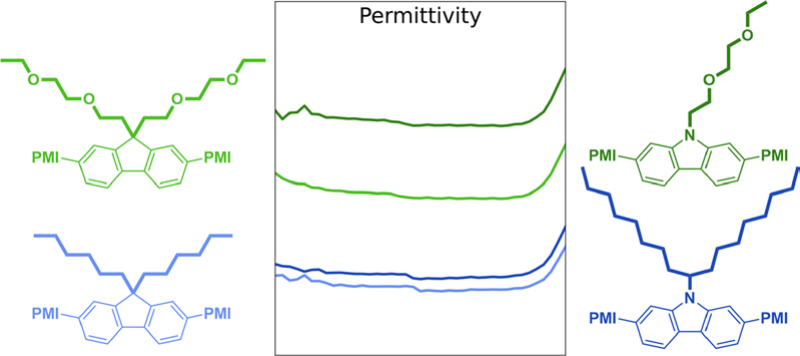

**Supplementary Information:**

The online version contains supplementary material available at 10.1007/s00706-022-02956-2.

## Introduction

Organic solar cells (OSCs) exhibit many innovative features, such as flexible and lightweight design, strong light absorption, possible semi-transparent fabrication, roll-to-roll processing and competitive power conversion efficiency (PCE) [1–4]. The research on novel non-fullerene acceptors displays an important pillar in this successful development of OSCs and led to highly efficient solar cells approaching now PCEs of almost 20% [5, 6]. Typical benefits of non-fullerene acceptors are the tunability of their optical properties and energy levels as well as their crystallization behaviour. Moreover, non-fullerene acceptor organic solar cells can simultaneously reveal low voltage losses and high photocurrents, which is crucial for obtaining high PCEs [5]. Perylene monoimide (PMI)-based acceptor materials possess many desired properties for OSCs including strong visible absorption, a low-lying lowest unoccupied molecular orbital (LUMO) energy level and a high photostability [7]. Furthermore, they are available via efficient synthetic routes, which also offer numerous options for modifications [8]. For these reasons, perylenes are widely used in various optoelectronic applications, such as in organic light-emitting diodes, OSCs and fluorescent probes [7, 9].

Popular designs for PMI-based OSC materials are acceptor–donor-acceptor (A-D-A) molecules, comprising two-electron-withdrawing PMI end-groups and an electron-donating linker. By adjusting the linker, it is possible to selectively fine-tune the highest occupied molecular orbital (HOMO) levels, aggregation behavior and charge mobilities of the molecules [10]. Therefore, a variety of linker molecules have been studied so far [7, 11, 12]. In recent studies, we have investigated PMI-linker-PMI acceptors using alkylated fluorene, silafluorene, indenofluorene and carbazole as linkers. Solar cells based on these acceptors revealed high open-circuit voltages of up to 1.4 V and average PCEs of 4.3–5.1% [13, 14].

However, a limitation of organic semiconductors is their poor dielectric permittivity *ε* (typical values of the relative permittivity *ε*_r_ are 3–4 for organic vs. 10–15 for inorganic semiconductors) [15, 16]. This manifests in wide-ranging negative effects within OSCs, such as a high exciton binding energy, a high geminate recombination rate, limited fill factor (FF) and current densities [17]. This altogether limits the possible performance of perylene dyes in OSCs, regardless of their numerous attractive properties.

Here, we report the synthesis of two new PMI-based A-D-A acceptors, PMI-[**C-OEG**] and PMI-[**F-OEG**] (Scheme 1) with carbazole and fluorene as respective linking unit. Both linkers bear oligo ethylene glycol (OEG) side chains, which should increase the permittivity of the molecules by introducing freely-rotating, polarizable dipoles that are able to shield charges from each other [17–19]. To investigate the influence of polar side chains on the dielectric permittivity and photovoltaic performance, the newly developed materials were analyzed and compared with analog compounds bearing simple alkyl chains, PMI-[**C-ALK**] and PMI-[**F-ALK**] (Scheme 1), which we introduced in a previous work [14].

**Figure:**
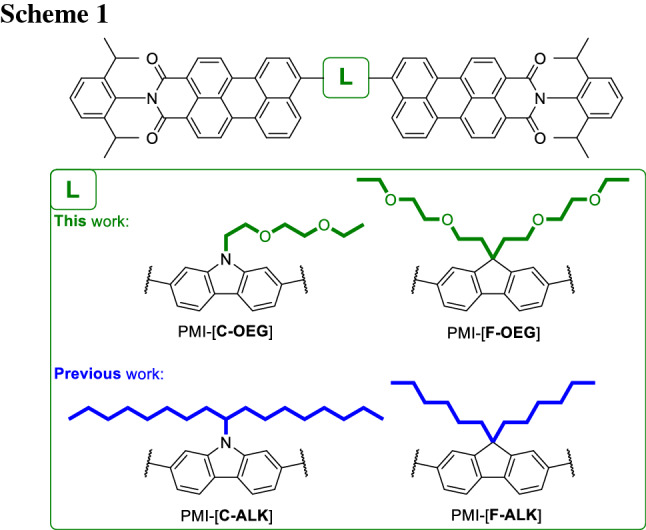

## Results and discussion

Scheme 2 depicts the synthetic pathway to the target molecules PMI-[**C-OEG**] and PMI-[**F-OEG**]. The commercially available 2-(2-ethoxyethoxy)ethanol (**1**) was treated with PBr_3_ to give the primary bromide (**2**). The product could easily be separated by distillation. It was then reacted with freshly recrystallized carbazole- (**3**) or fluorene- (**4**) 2,7-dibromides. Both products (**5** and **6**) were isolated by column chromatography (eluent petrol ether: ethyl acetate or toluene: acetone, respectively) and obtained in adequate yields (68% and 63%, respectively). These building blocks were then used in a subsequent Suzuki coupling with perylene monoimide boronic acid pinacol ester (**7**) which was prepared from perylene dianhydride in a 3-step reaction according to literature procedures [20]. This gave the desired PMI-[**C-OEG**] and PMI-[**F-OEG**]. The yields of both reactions were low to moderate (12% to 51%, respectively). This can be attributed to slow rates of the desired product formation, which left time for typical side reactions of the Suzuki coupling reaction [21]. Homocoupling to PMI-PMI and protodeboronation of **7** were observed by TLC and ^1^H NMR spectroscopy [20]. Consequently, the possible yield is reduced twofold, first because of the reaction itself, and second because of difficult purification. Also, column chromatography turned out to be challenging due to the ambivalent nature of the acceptor materials (polar side chains on a non-polar aromatic system). Despite these obstacles, both products could be obtained after multiple column purification processes and final recrystallization steps. The identity and purity of the prepared compounds were determined by NMR and FT-IR spectroscopy and HR-MS.

**Figure:**
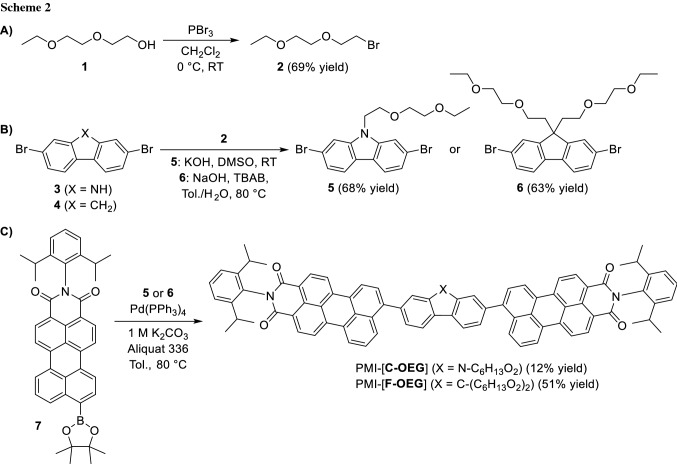

**Fig. 1:**
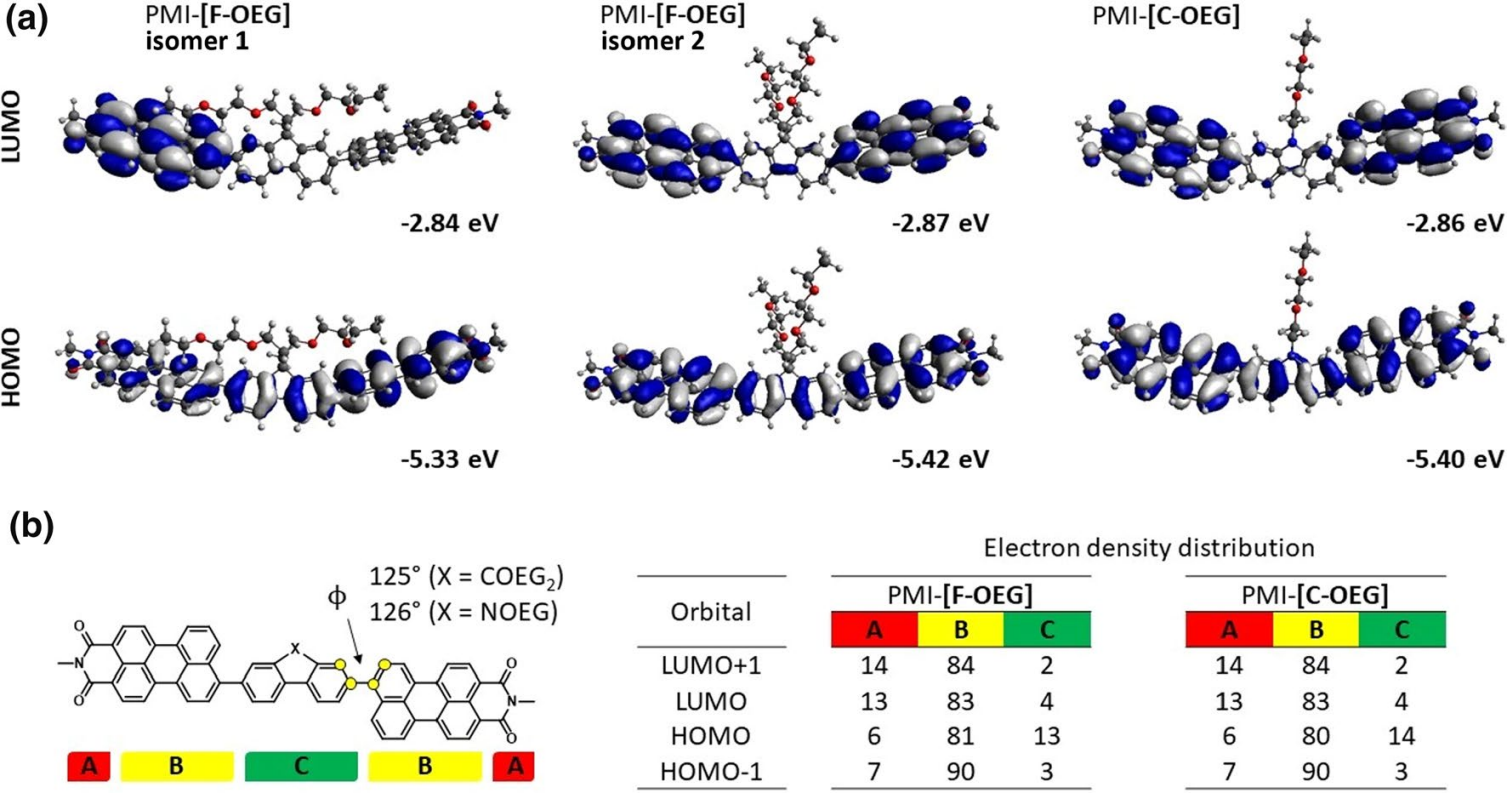
**a** Frontier molecular orbitals and their energies for two PMI-[**F-OEG**] isomers with a different side chain orientation and for PMI-[**C-OEG**]. **b** NAO analysis of electron density distribution in both acceptors. The torsion angle between the perylene core and the linker is given above the structure on the left side. Full side chains were used in the calculations but the 2,6-*i*-Pr-phenyl group on the imide nitrogen atom was substituted by methyl groups. Computations were done on a B3LYP GD3 level of theory using 6-31G(d,p) basis set

To determine the influence of the new side chains on the electronic and geometric properties of the molecules, we carried out DFT simulations on both molecules. Ground state geometry optimization was performed on B3LYP-GD3 level of theory using 6-31G(d,p) basis set as implemented in Gaussian09 package [22]. Primarily, we addressed two questions: First, what is the orientation of the perylene monoimides relative to each other and second, how are the side chains orientated. We found that the anti-isomer (perylene monoimides antiperiplanar) is slightly lower in energy than the syn isomer. Simultaneously, the orientation of the side chains has a larger impact on the overall energy. It is more favorable to have the side chains located along the perylene core (Fig. 1, PMI-[**F-OEG**] isomer 1) than to have the side chains pointing away from the aromatic system (Fig. 1, PMI-[**F-OEG**] isomer 2), which rises the energy of the molecule by 56 kJ mol^−1^ (or 50 kJ mol^−1^ if the polarizable continuum model with CHCl_3_ as solvent is used in the calculations). A model compound PMI-[**F**-**ALK**] with two hexyl side chains shows only a minimal change in energy when the side chains are located along the π-system or are pointing away from it (∆*E* =  + 2 kJ mol^−1^ in case of the former, see also Supplementary Information (SI) Fig. S15). Clearly, the larger differences for PMI-[**F-OEG**] must rise from the interaction of the free electron pairs of the oxygen atoms in the OEG side chains with the aromatic core. The LUMO and, particularly, the HOMO energy levels are upshifted for the isomer in which the OEG chains are located along the perylene core. Also, the electron distribution in these orbitals is slightly different if compared to PMI-[**F-OEG**] with the side chains pointing away from the perylene core (Fig. 1a).

Next, we performed time-dependent (TD)-DFT computations to probe the excitation properties of these molecules. It was found that the PMI-[**F-OEG**] isomer 1, in which the side chains are located along the perylene core, should have a strong absorption peak at 562 nm, followed by less intensive transitions at 533, 517, and 497 nm (Table 1). At the same time, if the side chains are located away from the perylene core, only two strong transitions are predicted in the visible range, one at 548 and a second at 501 nm. Experimental spectra (in solution) show a maximum at 530 nm and a shoulder at 507 nm. The calculation in which the OEG side chains are located away from the perylene core are in a much better agreement with the experimental observations. This is strong evidence that in solution and at room temperature, the OEG chains are not located along the perylene π-system despite the predicted energetical minimum. Therefore, analysis of the electron density in PMI-[**F-OEG**] and PMI-[**C**-**OEG**] were performed for structures in which the side chains are located away from the perylene core.

**Table 1:** Calculated excitation properties of PMI-[**F-OEG**] and PMI-[**C**-**OEG**]

Compound	State	Orbitals^a^	eV	nm	Oscillator strength
PMI-[**F**-**OEG**]					
isomer 1	S_1_	H $\to$ L^b^	2.21	562	0.85
	S_2_	H $\to$ L + 1	2.33	533	0.53
	S_3_	H-1 $\to$ L	2.40	517	0.15
	S_4_	H-1 $\to$ L + 1	2.49	497	0.20
PMI-[**F**-**OEG**]					
isomer 2	S_1_	H $\to$ L	2.26	548	1.34
	S_4_	H-1 $\to$ L + 1	2.47	501	0.47
PMI-[**C**-**OEG**]	S_1_	H $\to$ L	2.25	550	1.32
	S_4_	H-1 $\to$ L + 1	2.47	502	0.46

Calculations were done on a B3LYP-GD3 level of theory using 6-31G + (d,p) basis set

^a^for the PMI-[**F-OEG**] isomer 1, only the main orbitals involved in the excited state are shown (see **SI Table S1** for a full list)

^b^H stands for HOMO, L stands for LUMO

In both compounds a similar torsion angle between the perylene units and the linker are found (125–126°). From the molecular orbital isosurfaces (Fig. 1a), it can be seen that a significant shift in the electron density from the linker to the perylene core happens upon transition from HOMO to LUMO orbitals. Natural atomic orbital (NAO, [23]) analysis allows to quantify this change. In order to do that, we separated the molecule in three units: A – imide group, B – perylene core, C – linker (Fig. 1b). It was found that in both compounds, the HOMO–LUMO transition has a charge transfer character, as the electron density on the linker (part “C”, green) changes from 13 to 14% in HOMO to 4% in LUMO, which supports the A-D-A structure in the molecules.

To further explore the orientation of the side chains, we performed geometry optimization of a triad consisting of PMI-[**F-OEG**] isomer 1 or isomer 2 with two perylene monoimides placed on top. Calculations of such large systems using DFT methods would be expensive and time-consuming, thus we used the HF-3c method implemented in Orca 4.2 [24]. We found that the structure in which OEG side chains are pointing away from the perylene core can have much stronger intermolecular interactions between π-scaffolds and thus have a lower energy (see also SI Fig. S15). However, these results should be viewed only as an estimation. For more detailed investigation, computations would need to be supported by spectroscopic measurements.

**Fig. 2:**
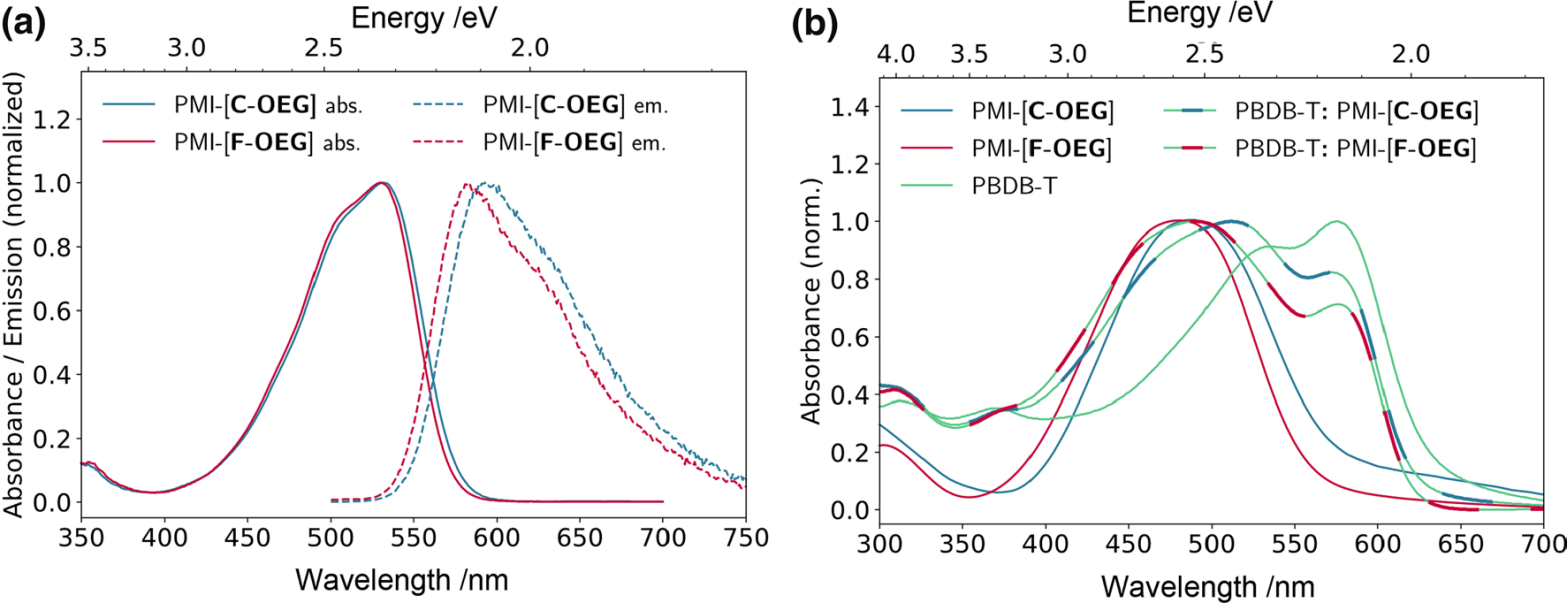
Optical characterization: **a** Absorbance (solid) and emission (dashed) spectra in chloroform (both at 0.01 mg cm^−3^, emission excited at 485 nm) **b** solid-state absorbance of pristine acceptors, PBDB-T and the active layer blends (donor–acceptor weight ratio 1:1)

Moreover, the optical properties of both solutions and thin films were investigated by UV–Vis as well as fluorescence spectroscopy. Both acceptors have their solution absorption maximum located around 530 nm (Fig. 2a), with the molar absorption coefficients of 9.2 × 10^4^ dm^3^ mol^−1^ cm^−1^ in both cases (Table 2). Also, both compounds exhibit a second absorption peak at shorter wavelength (507 nm). The first absorption peak is a HOMO–LUMO transition (Table 1 in DFT section) and has a charge transfer character (from the linker to perylene monoimide). The second absorption peak is a HOMO-1 to LUMO + 1 transition, which can be identified as a locally excited state on the perylene monoimide (see the MO electron density changes in Fig. 1b).

**Table 2:** Characteristic optical quantities of the new acceptors

Compound	${\lambda }_{\mathrm{max}}^{\mathrm{sol}}$/nm	${\lambda }_{\mathrm{max}}^{\mathrm{tf}}$/nm	${\varepsilon }_{\mathrm{max}}^{\mathrm{sol}}$/dm^3^ mol^−1^ cm^−1^	${E}_{\mathrm{g}}^{\mathrm{sol }a}$/eV	${E}_{\mathrm{g}}^{\mathrm{tf }b}$/eV	$\Delta \overline{\nu }$/cm^−1^	${\Phi }_{\mathrm{Fl}}$/%
PMI-[**C-OEG**]	532	487	9.2 × 10^4^	2.21	2.14	1930	64 ± 2
PMI-[**F-OEG**]	530	482	9.2 × 10^4^	2.23	2.21	1770	72 ± 5

^a^Determined from the intersection of the absorbance and emission curves

^b^Determined from the absorbance onset of pristine thin films (spin coated from a 10 mg cm^−3^ chloroform solution onto glass plates without post-treatment)

The fluorescence spectra show emission maxima at 593 nm and 585 nm with Stokes shifts of 61 nm (1930 cm^−1^) and 55 nm (1770 cm^−1^) for PMI-[**C-OEG**] and PMI-[**F-OEG**], respectively. The reference compounds show similar Stokes shifts, which supports the idea that the side chains do not significantly influence the optical properties [14]. In addition, both acceptors have fluorescence quantum yields of 64% and 72% for PMI-[**C-OEG**] and PMI-[**F-OEG**], respectively. This suggests that additional non-radiative relaxation pathways are present.

In thin films (Fig. 2b), both acceptors show one broad absorption peak at approx. 485 nm. PMI-[**C-OEG**] has a more red-shifted absorption maximum and onset, indicating a stronger ordering of the molecules in the solid-state compared to PMI-[**F-OEG**] due to the fact that the carbazole derivative has only one side chain instead of two allowing for a denser packing. The same observation was made for the compounds with alkyl side chains [14]. The solid-state absorption maximum of PBDB-T (PBDB-T: poly[[4,8-bis[5-(2-ethylhexyl)-2-thienyl]benzo[1,2-*b*:4,5-*b′*]dithiophene-2,6-diyl]-2,5-thiophenediyl[5,7-bis(2-ethylhexyl)-4,8-dioxo-4*H*,8*H*-benzo[1,2-*c*:4,5-*c′*]dithiophene-1,3-diyl]]) (Fig. 4b), the conjugated polymer chosen to be used as donor for the investigation of the photovoltaic properties of PMI-[**C-OEG**] and PMI-[**F-OEG**], is located at 575 nm. Thus, PBDB-T blends with both new acceptor materials cover a large part of the visible light spectrum (Fig. 2b).

**Fig. 3:**
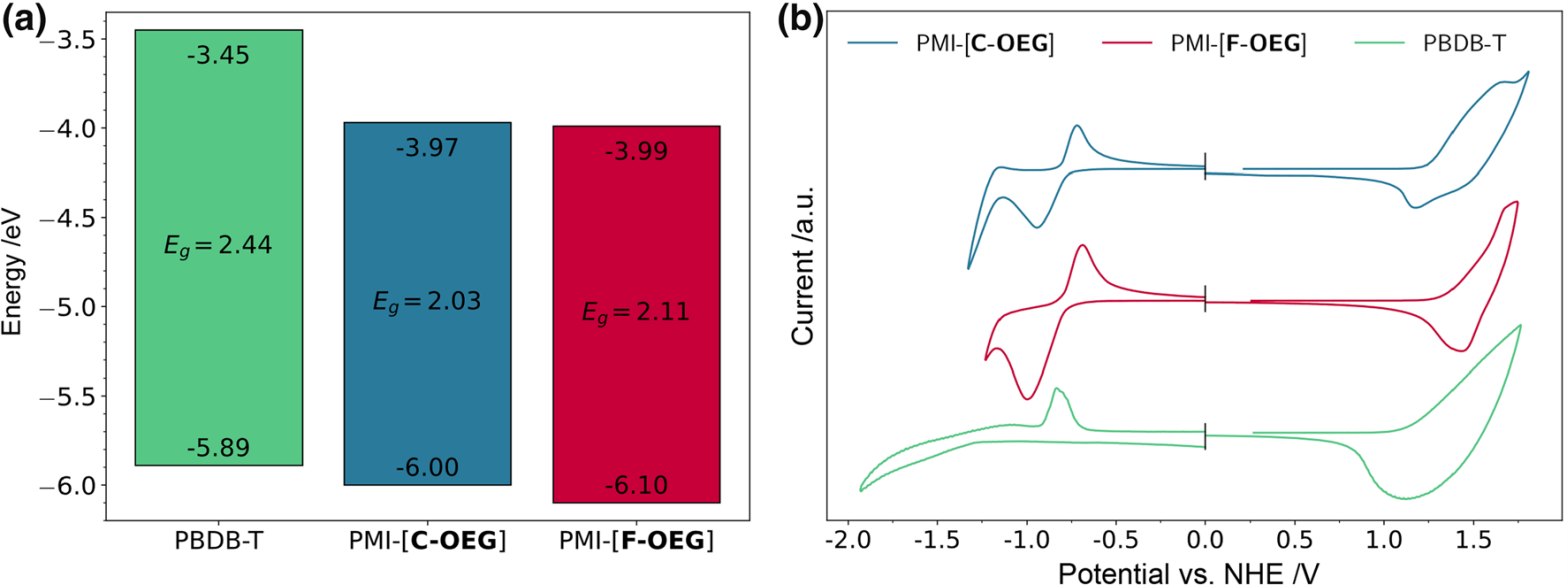
**a** Frontier orbital energies extracted from CV measurements, **b** CV measurements of the pristine acceptors and PBDB-T (3-electrode setup, drop-cast films on Pt disc working electrode, Pt counter electrode, Ag/AgCl reference electrode, 0.1 M TBAPF_6_ in acetonitrile as electrolyte, scan speed 50 mV s^−1^)

Cyclic voltammetry (CV) measurements of drop-casted thin films (Fig. 3) reveal the influence of the OEG side chain on the frontier orbital energies. PMI-[**C**-**OEG**] has HOMO and LUMO energies of − 6.00 eV and − 3.97 eV, respectively. Compared to the reference compound PMI-[**C**-**ALK**] (HOMO/LUMO − 6.16 / − 3.94 eV [14]), the HOMO is elevated by 0.16 eV, while the LUMO remains relatively similar. PMI-[**F**-**OEG**] (HOMO/LUMO  – 6.10 /  – 3.99 eV), on the other hand, has similar values as PMI-[**F**-**ALK**] (HOMO/LUMO − 6.12 / − 3.93 eV).

PMI-[**C**-**OEG**] shows a slightly higher HOMO than PMI-[**F**-**OEG**], while revealing similar LUMO energy levels. This is likely due to the electron rich nature of the carbazole linker. The values promise a good fit with PBDB-T (HOMO/LUMO  – 5.89 /  – 3.45 eV) as donor polymer.

To quantify the influence of the OEG chains on the dielectric permittivity of the new compounds, we conducted impedance spectroscopy measurements. To that end, capacitor devices were fabricated consisting of a polystyrene:NFA layer sandwiched between an ITO and an Ag electrode. The investigated acceptors were dispersed in a polystyrene matrix (1:1, w:w) to obtain homogeneous films. This fabrication method was needed to prevent electrical shorting of the devices. Three devices were built and measured for each acceptor (SI Fig. S20a, b). The relative permittivity values of the neat materials (SI Fig. S20c) were then calculated from the frequency-dependent impedance values via the Maxwell–Garnett equation (shown in the experimental section [23]).

**Fig. 4:**
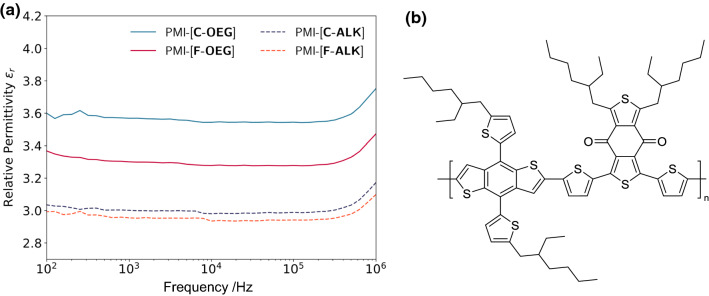
**a** Frequency-dependent relative permittivity *ε*_r_ of new and reference compounds (averaged from three devices, respectively), **b** molecular structure of PBDB-T

Below 10^2^ Hz, strong interferences occurred, especially at 50 Hz, which corresponds to the standard AC power supply frequency. Hence, only the frequency interval from 10^2^ to 10^6^ Hz was considered. Within this frequency interval, both acceptors show an increased relative permittivity compared to their reference compounds (Fig. 4a). Exemplary at 10^6^ Hz, *ε*_r_ of PMI-[**C**-**OEG**] increased by 18% from 3.17 to 3.75. Similarly, *ε*_r_ of PMI-[**F**-**OEG**] increased by 12% from 3.10 to 3.47. As general trend, but much more pronounced within the OEG acceptors, the carbazole-based acceptors show a higher permittivity than the fluorene-based acceptors.

To determine the thermal stability and annealing behavior of the new compounds, we further conducted thermogravimetric analyses (SI Fig. S16-S19). Both compounds show a high thermal stability. The degradation starts at approx. 440 °C for PMI-[**C-OEG**] and approx. 400 °C for PMI-[**F-OEG**]. Interestingly, PMI-[**C-OEG**] loses 2.5% mass at 100–200 °C. This corresponds to the evaporation of toluene, which appears to incorporate into the crystal structure in a molar ratio of 1:3 upon recrystallization (confirmed by ^1^H NMR spectroscopy). The glass transition temperatures are at 276 °C for PMI-[**C-OEG**] and 222 °C for PMI-[**F-OEG**] (SI Fig. S19).

**Fig. 5:**
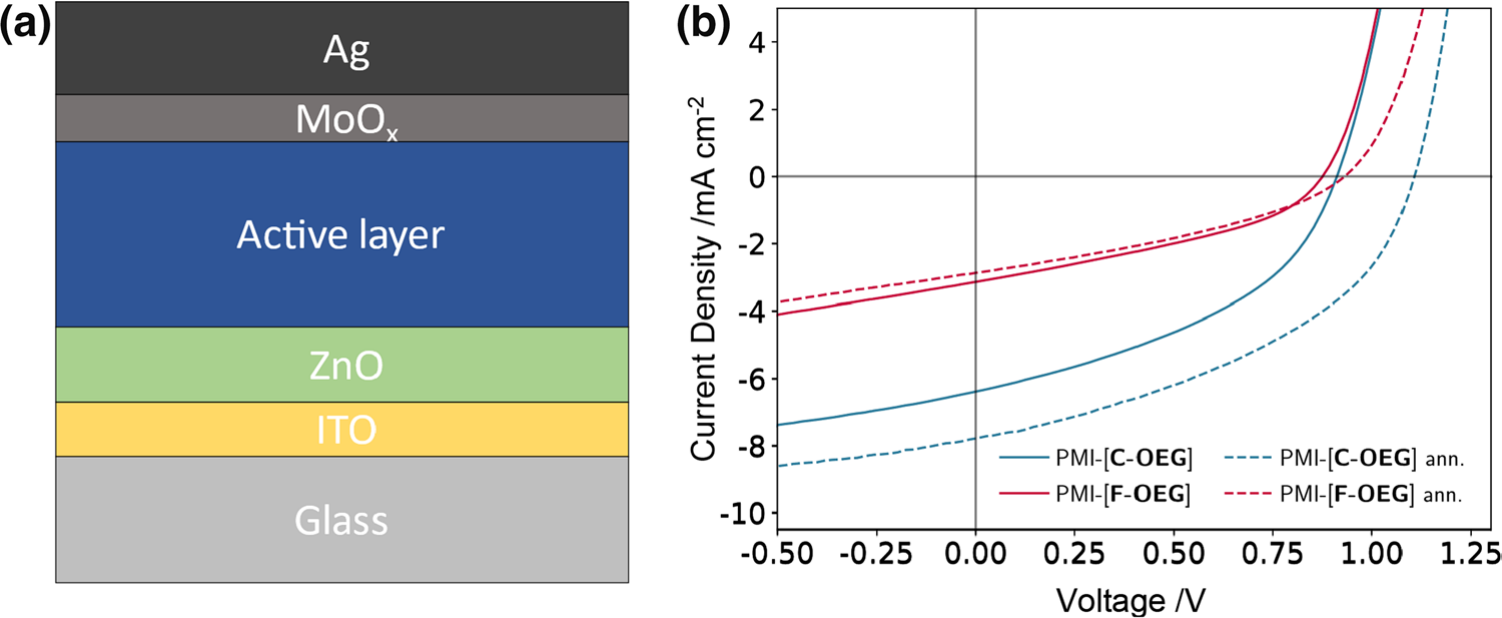
**a** Device architecture of the fabricated inverted OSCs; **b** typical *JV* curves of OSCs containing the new acceptors PMI-[**C-OEG**] (blue) and PMI-[**F-OEG**] (red), both with PBDB-T as donor, without and with (**ann.**) thermal annealing

Next, we investigated the photovoltaic properties of both new acceptors. We incorporated PMI-[**C-OEG**] and PMI-[**F-OEG**] as acceptor into OSCs using an inverted device architecture, comprising indium tin oxide (ITO)/ZnO/PBDB-T:acceptor/MoO_x_/Ag (Fig. 5a, details of the fabrication process can be found in the experimental section). Without annealing the absorber layer, the OSCs based on PMI-[**C-OEG**] and PMI-[**F-OEG**] achieve average PCEs of 2.53 ± 0.15% and 1.21 ± 0.06%, respectively (all values are listed in Table 3, *JV* curves are depicted in Fig. 5b). The PMI-[**C-OEG**]-based OSCs show an improved FF as well as *J*_SC_, which is in line with the higher to equal external quantum efficiency over the whole scanning range (EQE spectra and *JV* curves of the used OSCs are shown in Fig. S21). Moreover, the increased photocurrent contribution of the acceptor phase in these devices indicates a higher efficiency of PMI-[**C-OEG**] in the charge generation/separation process.

**Table 3:** Photovoltaic parameters of the OSCs with PBDB-T:acceptor blends with and without annealing (ann.) for 5 min, and a weight ratio of 1:1

Acceptor	ann.	*V*_OC_ /V	*J*_SC_ /mA cm^−2^	FF /%	PCE /%
PMI-[**C-OEG**]	210 °C	1.09 ± 0.01	7.82 ± 0.53	44 ± 1	3.71 ± 0.20
	RT	0.92 ± 0.01	6.37 ± 0.34	43 ± 1	2.53 ± 0.15
PMI-[**F-OEG**]	170 °C	0.98 ± 0.04	3.09 ± 0.21	36 ± 1	1.09 ± 0.07
	RT	0.87 ± 0.02	3.76 ± 0.22	37 ± 1	1.21 ± 0.06

The average values are calculated from the 5 best cells, respectively

After annealing of the absorber layers at 170 and 210 °C for 5 min, the *JV* curves of both acceptor-type OSCs (Fig. 5b) show quite different characteristics. On the OSCs based on PMI-[**F-OEG**], the annealing showed only little impact. The average PCE is 1.09 ± 0.07% with a slight reduction of *J*_SC_ and increase of *V*_OC_, compared to their untreated counterparts. The performance of OSCs with the PMI-[**C-OEG**] acceptor revealed an improved PCE of 3.71 ± 0.20%, with both an increased *V*_OC_ of 1.09 ± 0.01 V and *J*_SC_ of 7.82 ± 0.53 mA cm^−2^. This setup led to the highest observed PCE in this study of 3.92% (*V*_OC_ 1.10 V, *J*_SC_ 8.65 mA cm^−2^, FF 45%). The reason for the higher PCE of the carbazole-based OSCs lies most likely in the different crystallization behaviour of the two new acceptors. With only one side chain and a planar sp^2^-hybridized nitrogen atom on the linker, we assume that PMI-[**C-OEG**] shows a higher tendency to form pure, crystalline structures in the BHJ upon annealing. In contrast, the two side chains and sp^3^-hybridized carbon atom in PMI-[**F-OEG**] likely lead to both, a reduced crystallization tendency and potentially a better miscibility with the donor polymer PBDB-T. This would also explain the fact that fluorene-based OSCs show only little response to annealing.

In an identical device setup with the same donor, the reference compounds PMI-[**C-ALK**] and PMI-[**F-ALK**] show average PCEs of 4.45 ± 0.36% and 4.34 ± 0.37%, respectively [14]. This reveals that the new OEG based acceptors could not outperform the reference compounds, despite their improved dielectric properties. Studies on related approaches to increase the permittivity of OSC absorber materials have reported similar behaviour, which was generally ascribed to a less ideal bulk heterojunction morphology formed by the OEG side chain containing materials [17, 25, 26]. The herein presented experimental results indicate a similar behaviour of the new OEG side chain bearing acceptors.

**Fig. 6:**
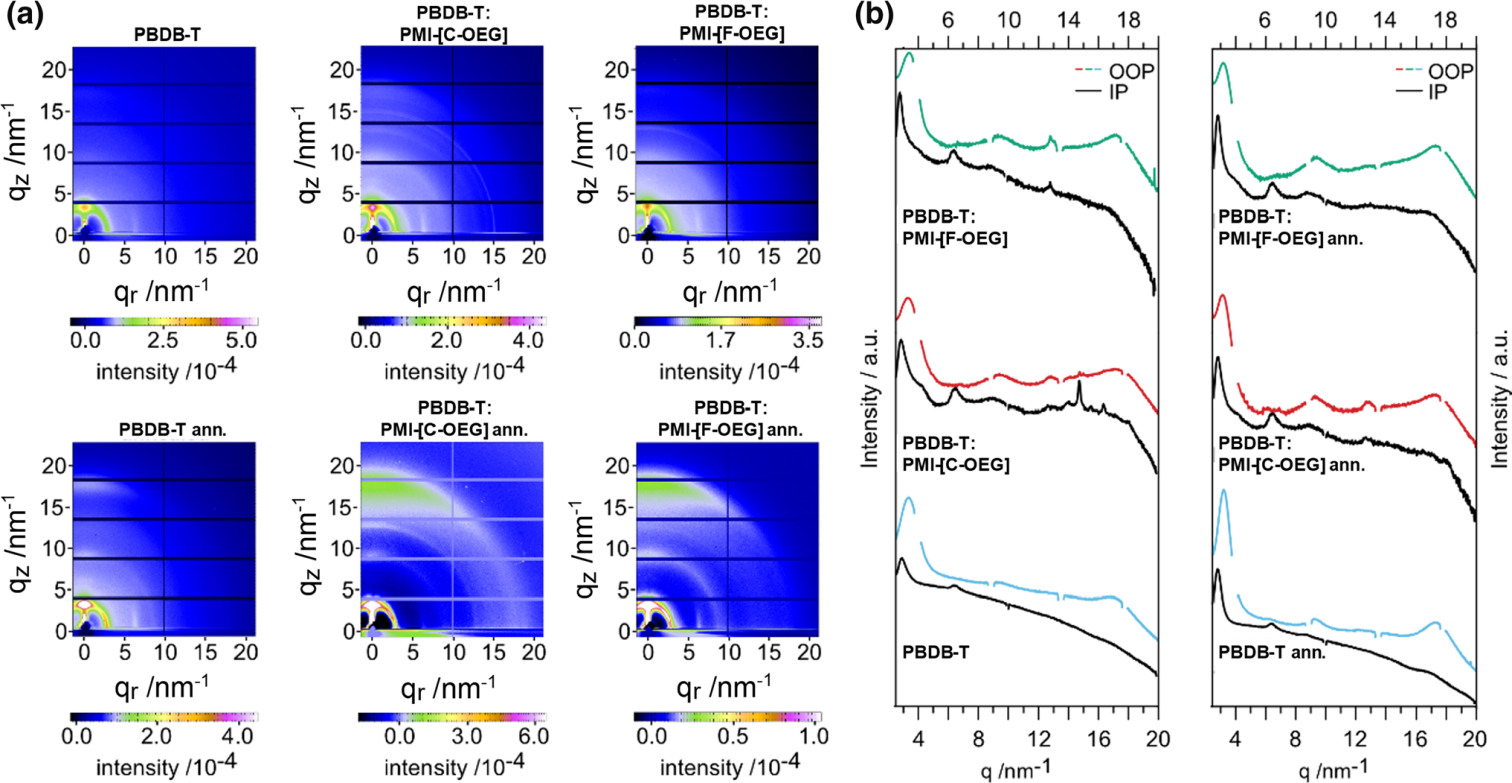
2D-GIWAXS characterizations of PBDB-T and the PBDB-T/PMI-[**C-OEG**] and PBDB-T/PMI-[**F-OEG**] blends: **a** GIWAXS images of as cast (top) and annealed (160 °C, 5 min) films (bottom), **b** line-cuts in in-plane (IP) and out-of-plane (OOP) direction of as cast (left) and annealed films (right)

To investigate the molecular packing of donor and acceptor in the bulk heterojunction thin films, we conducted grazing-incidence wide-angle X-ray scattering (GIWAXS) measurements of the pristine donor film and D-A blends before and after annealing at 160 °C. In the GIWAXS images (Fig. 6a) of the thin films without annealing as well as in the corresponding line cuts in in-plane and out-of-plane direction (Fig. 6b), only weak features are visible. After annealing, the crystallinity as well as the preferred orientation is increased. In the GIWAXS image of the pristine polymer (PBDB-T) film, the most significant features are the (010) diffraction peak in the out-of-plane direction at ~ 17.2 nm^−1^, corresponding to a π-π stacking distance of 0.37 nm and the (100) lamellar diffraction peak at 3.2 nm^−1^ visible in the out-of-plane direction with a semicircle-like extension to the in-plane direction, originating from a lamellar d-spacing with a distance of 2.0 nm. Moreover, a weaker feature at 9.3 nm^−1^ in out-of-plane direction (300) and one at 6.4 nm^−1^ in in-plane direction (001), which is characteristic for a high in-plane orientation of the polymer backbone, are observed. This pattern is typical for a preferential face-on orientation of PBDB-T relative to the substrate surface [27].

In the blend films, additional features stemming from the acceptor are observed in the GIWAXS images. The signals are similar to the ones of PMI-[**C-ALK**] and PMI-[**F-ALK**] [14] and are characteristic for a preferential face-on orientation of the molecules. In both blends, the most pronounced additional signal in the in-plane direction is the shoulder at 4.2 nm^−1^ (corresponding to a distance of 1.5 nm), which is more defined in the PMI-[**C-OEG**] film. In the out-of-plane direction, the diffraction peak at 17.2 nm^−1^ is more intense and slightly broadened in the blend films because of a convolution of the donor and acceptor contributions. Interestingly, compared to the pristine polymer film, this peak is not shifted to higher q-values as it was observed in blends of PBDB-T and PMI-[**C-ALK**] or PMI-[**F-ALK**] [14]. This supports the conclusion that the π–π stacking distance of PMI-[**C-OEG**] or PMI-[**F-OEG**] in the blends is slightly higher (approx. 0.37 nm) compared to their analogs bearing alkyl side chains (approx. 0.35 nm). The increased stacking distance might have adverse effects on the charge carrier transport in the OEG side chain-bearing NFAs and therefore also on the solar cell performance [28].

The signal at 8.7 nm^−1^, which might stem from the semicircular extension of the corresponding peak in out-of-plane direction is also present in the pristine polymer film. In addition, in the PMI-[**C-OEG**] sample, peaks are observed at 6.2 and 12.7 nm^−1^ in out-of-plane direction and the latter also in the in-plane direction. The sharp in-plane peaks observed between 13.7 and 16.7 nm^−1^ in the non-annealed PMI-[**C-OEG**] film are ascribed to inhomogeneities in the film due to not fully dissolved acceptor crystals in the solution before coating the film.

Overall, the acceptor component in the PMI-[**C-OEG**] blend film appears to be more crystalline than in the PMI-[**F-OEG**] sample, in particular in the annealed films, as the peaks at 6.2 and 12.7 nm^−1^ in out-of-plane direction are more pronounced and the shoulder at 4.2 nm^−1^ in in-plane direction is more defined. Such a behavior is in good agreement with the presented DFT calculations and could explain the higher photocurrents observed in the PMI-[**C-OEG**] based solar cells.

## Conclusion

In conclusion, we synthesized two new perylene monoimide-based acceptors, PMI-[**C-OEG**] and PMI-[**F-OEG**], bearing flexible and polar OEG side chains.

Both compounds show an increased dielectric permittivity over the whole measured frequency interval, compared to their alkylated analogues. Specifically, the relative permittivity (at 10^6^ Hz) increased by 18% from 3.17 to 3.75 for the carbazole-based acceptor PMI-[**C-OEG**] and by 12% from 3.10 to 3.47 for the fluorene-based acceptor PMI-[**F-OEG**]. Furthermore, both compounds show well-suited energy levels and optical bandgaps to be used as acceptor materials in organic solar cells. Despite their improved dielectric properties, unfavourable changes in the donor–acceptor morphology due to the introduction of the polar side chains and less pronounced π–π stacking of the acceptor, seem to be a major factor precluding that the increased permittivity is also reflected in improved solar cell efficiencies.

However, these results further support that the substitution of alkyl chains with polar OEG chains is a simple and straightforward method to improve the dielectric properties of acceptors for OSCs. For future research on this topic, we find it crucial to expand the focus on the donor polymer as well, since the ideal interplay of both materials is indispensable for efficient solar cells.

## Experimental

All reagents and solvents were purchased from commercially available sources (Sigma Aldrich, Lumtec, abcr, VWR, Roth) and used as received, unless otherwise stated. 2,7-Dibromofluorene and 2,7-dibromo-9*H*-carbazole were dried in vacuum over CaCl_2_ before use. Dry CH_2_Cl_2_ was prepared by distillation over P_4_O_10_. Dry THF was prepared by running the solvent through an automated aluminium oxide column.

**Column chromatography** was done manually using normal phase silica gel with a pore size of 60 μm.

**Geometry optimisation and TD-DFT** calculations of single molecules were performed in the Gaussian09 program package [22], on B3LYP level of theory, using GD3 [29] empirical dispersion correction. For ground state calculations, the 6-31G(d,p) basis set was used. The local minima reached upon geometry optimisation were confirmed by frequencies calculations. Results were visualised using Avogadro. NAO analysis [23] was done using Multiwfn [30]. For TD-DFT calculations, the 6-31G + (d,p) basis set was used and the first 10 excited states were calculated. Geometry optimisation of triads (PMI-[**F-OEG**] stacked with two perylene monoimides) were done with HF-3c method in the Orca 4.2 program package [24, 29, 31–33].

**NMR spectra** were recorded on a Bruker Avance 300 MHz and a Varian Inova 500 MHz. Deuterated solvents were purchased from EurisoTop GmbH. The spectra were referenced to the internal TMS signal or the residual solvent signal.

**Mass spectra** were recorded on a Micromass TofSpec 2E time-of-flight mass spectrometer from Waters. As matrices, dithranol or *trans*-2-[3-[4-(*tert*-butyl)phenyl]-2-methyl-2-propenylidene]malononitrile (DCTB) were used. All spectra were externally calibrated with a polyethylene glycol standard. The spectra were interpreted using the MassLynx Software V3.5 from Micromass/Waters.

**TGA measurements** were done on a STA Jupiter 449C from Netzsch in aluminium crucibles under helium atmosphere. Measurements were performed from 20 to 550 °C with a scan rate of 10 °C min^−1^. DSC measurements were done on a DSC 8500 from Perkin Elmer under nitrogen atmosphere from 30 to 380 °C and a scan rate of 20 °C min^−1^. The second heating loop was used for the extraction of the glass transition temperature.

**UV–Vis spectra** in solution (0.01 mg cm^−3^ in chloroform, in 1 cm optical glass cuvettes from Hellma) and thin film (drop cast from 10 mg cm^−3^ chloroform solution) were recorded on a Shimadzu UV-1800 spectrometer.

**Fluorescence spectra** in solution (approx. 0.01 mg cm^−3^ in chloroform) were measured on a FluoroLog 3 spectrofluorometer from Horiba Scientific Jobi Yvon together with a R2658 photomultiplier from Hamamtsu. The spectra were recorded from 500 to 800 nm with a slit width of 1*.*0 nm and excitation wavelength of 485 nm. Lumogen orange from Kremer Pigmenete GmbH & Co.KG was used as reference compound for the relative fluorescence quantum yield calculations. The quantum yields were calculated according to [34]

1 $${\Phi } = {\Phi }_{ref} \frac{{f_{ref} }}{f}\frac{I}{{I_{ref} }}\frac{{n^{2} }}{{n_{ref}^{2} }}$$

where *Φ*_*ref*_ is the fluorescence quantum yield of the used reference compound (for lumogen orange used 95% [35]), *I* is the integrated fluorescence photon flux, *n* is the refractive index of the solvent (chloroform), and *f* is the absorption factor at the excitation wavelength (calculated by *f* = 1-10^*−A*^ where *A* is the absorption at the excitation wavelength).

**FT-IR spectra** were recorded on a Bruker ALPHA-p FT-IR spectrometer from solid samples in an attenuated total reflection (ATR) setup. The spectra were interpreted on the software OPUS 7*.*5.

**CV** measurements were done with a Jaissle Potentiostat–Galvanostat IMP 88 PC-100 in a standard three-electrode setup with an Ag/AgCl wire as quasi-reference electrode and two Pt-plates as working and counter electrodes in 0.1 M tetrabutylammonium hexafluorophosphate (TBAPF_6_) in acetonitrile as electrolyte. The respective material was drop cast onto the working electrode on a hotplate at 50 °C in a nitrogen filled glove box. To avoid interference from trapped charges, oxidation and reduction were measured separately with freshly prepared films and a scan speed of 50 mV s^−1^. Fc/Fc^+^ was used as external standard. The orbital energies were calculated using [36]

2 $$E_{{{\text{HOMO}}}} = - \left( {4.75 + E_{{{\text{onset vs}}.{\text{NHE}}}}^{{{\text{ox}}}} } \right){\text{eV}}$$

3 $${\text{E}}_{{{\text{LUMO}}}} = - \left( {4.75 + {\text{E}}_{{{\text{onset vs}}.{\text{NHE}}}}^{{{\text{red}}}} } \right){\text{eV}}$$

The Fermi energy level of NHE vs. vacuum was taken as 4.75 eV [37], whereas the redox potential of Fc/Fc^+^ vs. NHE was taken as 0.64 V [38, 39].

**GIWAXS** measurements were done at the Austrian SAXS beamline at the synchrotron ELETTRA in Trieste, Italy. The setup used a photon energy of 8 keV, a Dectris Pilatus3 1 M detector at a sample distance of 294 mm and an incidence angle of 1.1°. The 2D images and 1D line-cuts were made using the software SAXS Dog [40]. For the measurements, the compound films were spin-coated onto silicon substrates (grade: prime, thickness: 625 ± 20 µm) from Siegert Wafer.

The **relative permittivity** of the pristine materials was calculated from impedance measurements of the fabricated capacitors. From the measured capacity values, the relative permittivity of the acceptor-PS blend (*ε*_eff_) was calculated using the classical equation of a parallel-plate capacitor

$$\varepsilon_{eff} = \frac{Cd}{{\varepsilon_{0} A}}$$

where *C* is the measured capacity of the diode, *d* and *A* the thickness and cross-section of the acceptor-PS film, respectively, and *ε*_0_ the permittivity of the vacuum (8.8542 × 10^12^ F m^−1^). Subsequently, the permittivity of the pristine acceptor (*ε*_NFA_) was calculated using the Maxwell–Garnett equation [41]

$$\varepsilon_{{{\text{NFA}}}} = \frac{{\frac{{2\varepsilon_{{\text{M}}} }}{{\delta_{{\text{i}}} }}\left( {\frac{{\varepsilon_{{{\text{eff}}}} - \varepsilon_{{\text{M}}} }}{{\varepsilon_{{{\text{eff}}}} + 2\varepsilon_{{\text{M}}} }}} \right) + \varepsilon_{{\text{M}}} }}{{1 - \frac{1}{{\delta_{{\text{i}}} }}\left( {\frac{{\varepsilon_{{{\text{eff}}}} - \varepsilon_{{\text{M}}} }}{{\varepsilon_{{{\text{eff}}}} + 2\varepsilon_{{\text{M}}} }}} \right)}}$$

where *δ*_i_ is the volume fraction of the acceptor (assumed 0.5 with density PS = density NFA), and *ε*_M_ is the permittivity of the PS matrix (measured *ε*_M_ = 2.44).

***J-V* curves** were recorded inside a glove box with a Keithley 2400 source meter connected to a LabView-based software. The active area of the solar cells was defined by illumination through a shadow mask (2.65 × 2.65 mm^2^). As light source, a Dedolight DLH400 lamp with a similar emission spectrum to AM1.5G was used at an intensity of 100 mW cm^−2^. Scans were done from 1.50 V to − 0.50 V (step width − 0.02 V, delay 100 ms, compliance 100 mA).

**External quantum efficiency (EQE) spectra** were measured with a Stanford Research Systems SR 830 DPS lock-in amplifier. Light source was a 75 W xenon lamp connected to a Multimode 4-AT monochromator from Amko. The light was chopped at a frequency of 30 Hz. The setup was calibrated with a silicon photodiode (818-UV/DB, Newport Corporation). The spectra were recorded from 380 to 900 nm with a step width of 10 nm.

**Capacitance measurements** were conducted on a ModuLab XM PhotoEchem optical and electrical measurement system from Solatron Analytical, AMETEK.

**Solar cell fabrication** was done in the following inverted architecture: ITO/ZnO/active layer/MoO_x_/Ag. For that, pre-patterned glass/ITO substrates (15 × 15 ×  1.1 mm^3^, 15 Ω sq^−1^) were cleaned by wiping them with acetone, sonication in a 2-propanol bath (40 °C, 30 min), blow-drying in a nitrogen stream and finally oxygen plasma etching (9 W, 3 min, FEMTO, Diener Electronics). The ZnO layer was prepared by a sol–gel process. As precursor solution, 500 mg of zinc acetate dihydrate was dissolved in 5 cm^3^ 2-methoxyethanol and 150 mm^3^ ethanolamine as stabilizer. The solution was stirred at least overnight and filtered with a 0.45 μm PTFE syringe filter just before deposition. The precursor solution was spin coated (4000 rpm, 30 s) and annealed (150 °C, 15 min) in ambient conditions. For the active layers, precursor solutions were prepared (D/A ratio 1:1 w/w, 20 mg cm^−3^ total concentration in chlorobenzene) in a glove box and stirred at 60 °C overnight. The layers were applied with varying spin coating parameters (1000 to 4000 rpm for 60 s, then drying at 5000 rpm for 5 s). Finally, the MoO_x_ (10 nm, 0.1–0.5 Å s^−1^) and Ag (100 nm, 0.1–2.0 Å s^−1^) layers were subsequently applied by thermal evaporation at high vacuum (< 1 × 10^–5^ mbar, thickness monitor: Inficon SQM-160 rate/thickness monitor) through a shadow mask to define the active area (3 × 3 mm^2^).

The **capacitors** for the impedance measurements were prepared as followed. The pre-patterned and cleaned ITO substrates for solar cells were also used to fabricate diode devices. As a first step, a 20 mg cm^−3^ solution of polystyrene (PS) in chloroform was stirred at RT overnight for full dissolution. Subsequently, the probe material was added to the PS solution to obtain a 40 mg cm^−3^ solution of a PS:acceptor blend with a 1:1 weight ratio. The blend solution was stirred for 1 h at 60 °C to fully dissolve the acceptor material, and was then spin coated onto the ITO substrate with 2400 rpm for 30 s under nitrogen atmosphere to obtain film thicknesses around 500 nm. Finally, a silver electrode was thermally evaporated on top of the PS:acceptor layer using a shadow mask.

### 1-Bromo-2-(2-ethoxyethoxy)ethane (2)

was prepared as described in Ref. [42]. A detailed procedure is enclosed in the SI. Yield 6.94 g (69%), light brown liquid, *R*_*f*_ = 0.84 (cyclohexane:acetone 1:1). The ^1^H NMR spectrum was found to be identical with literature.

### **2,7-Dibromo-9-[2-(2-ethoxyethoxy)ethyl]-9*H*-carbazole (5, C**_**18**_**H**_**19**_**Br**_**2**_**NO**_**2**_**)**

The procedure was adapted from literature [43]. 2,7-Dibromocarbazole (**3**, 1.17 g, 3.60 mmol) and 1.02 g crushed KOH (18.1 mmol) were dissolved in 15 cm^3^ DMSO. The flask was flushed with nitrogen for 5 min and 1.08 g **2** (5.24 mmol) was added. The mixture was stirred at RT for 19 h under nitrogen atmosphere. For workup, the mixture was extracted with 50 cm^3^ ethyl acetate. The combined organic phase was washed with 2 × 25 cm^3^ water, 25 cm^3^ brine and dried over Na_2_SO_4_. The solution was filtered and the filter residue rinsed with THF. Evaporation of the solvent under reduced pressure yielded light yellow crystals. Purification was done by flash chromatography (eluent toluene:acetone 99:1). Yield 1.09 g (68%); colourless solid; *R*_*f*_ = 0.20 (toluene:acetone 99:1); ^1^H NMR (300 MHz, (CD_3_)_2_SO): *δ* = 8.09 (d, ^3^*J*_HH_ = 8.3 Hz, 2H), 7.91 (s, 2H), 7.35 (dd, ^3^*J*_HH_ = 8.2 Hz, ^4^*J*_HH_ = 1.3 Hz, 2H), 4.56 (t, ^3^*J*_HH_ = 4.2 Hz, 2H), 3.76 (t, ^3^*J*_HH_ = 4.2 Hz, 2H), 3.41 (t, ^3^*J*_HH_ = 4.0 Hz, 2H), 3.30 (t, ^3^*J*_HH_ = 4.0 Hz, 2H), 3.24 (q, ^3^*J*_HH_ = 7.0 Hz, 2H), 0.96 (t, ^3^*J*_HH_ = 7.0 Hz, 3H) ppm; ^13^C NMR (75 MHz, (CD_3_)_2_SO): *δ* = 141.5, 122.1, 121.9, 120.7, 119.0, 113.0, 70.2, 69.2, 69.1, 65.6, 43.0, 15.0 ppm.

### **2,7-Dibromo-9,9-bis[2-(2-ethoxyethoxy)ethyl]-9*H*-fluorene (6)**

was prepared as described in Ref. [44]. A detailed procedure is enclosed in the SI. Yield 1.50 g (63%); colourless solid; *R*_*f*_ = 0.35 (petrol ether:ethyl acetate 5:1); ^1^H NMR (300 MHz, CDCl_3_): *δ* = 7.60–7.40 (m, 6H), 3.42 (q, ^3^*J*_HH_ = 6.8 Hz, 4H), 3.34 (t, ^3^*J*_HH_ = 4.3 Hz, 4H), 3.20 (t, ^3^*J*_HH_ = 4.3 Hz, 4H), 2.97 (t, ^3^*J*_HH_ = 7.2 Hz, 4H), 2.35 (t, ^3^*J*_HH_ = 7.2 Hz, 4H), 1.15 (t, ^3^*J*_HH_ = 6.8 Hz, 6H) ppm; ^13^C NMR (75 MHz, CDCl_3_): *δ* = 151.0, 138.5, 130.8, 126.8, 121.8, 121.3, 70.2, 69.8, 66.9, 66.7, 52.0, 39.6, 15.2 ppm.

### **2-(2,6-Diisopropylphenyl)-8-(4,4,5,5-tetramethyl-1,3,2-dioxaborolan-2-yl)-1*H*-benzo[10,5]-anthra[2,1,9-*def*]isoquinoline-1,3(2*H*)-dione (7)**

was prepared as described in Ref. [20]. A detailed procedure is enclosed in the SI. Yield 800 mg (59%); red solid; *R*_*f*_ = 0.85 (CH_2_Cl_2_). The ^1^H NMR spectrum was found to be identical with literature [19]. ^13^C NMR (75 MHz, CDCl_3_): *δ* = 164.2, 164.1, 145.9, 138.2, 138.0, 137.5, 137.4, 136.4, 132.2, 132.0, 131.9, 131.8, 131.2, 130.5, 129.6, 129.1, 127.9, 127.3, 127.0, 125.1, 124.1, 123.8, 122.9, 121.5, 120.9, 120.4, 84.4, 29.3, 25.1, 24.2 ppm; HR-MS (MALDI-TOF): *m/z* calcd. C_40_H_39_BNO_4_^+^ ([M + H]^+^) 608.2979, found 608.4222; FT-IR: $\overline{V}$ = 1699, 1661 (OCNCO imide) cm^−1^.

### General procedure for Suzuki coupling

The procedure was adapted from literature [45]. For the actual quantities used and purification details, please check below the general section. Compound **7** (2.4 equiv) and the respective linker (**5** or **6**, 1.0 equiv) were weighed into a 3-neck round bottom flask equipped with a stir bar, reflux condenser and nitrogen inlet connected to a Schlenk line. Toluene was added and the setup was flushed for 5 min with nitrogen. 1 M K_2_CO_3_ (11 equiv) and 1–2 drops of Aliquat 336 were added, upon which the setup was flushed with nitrogen for another 10 min. Then the catalyst [Pd(PPh_3_)_4_] (0.1 equiv) was added and the mixture was refluxed until TLC control indicated full conversion. Workup was done by diluting with CH_2_Cl_2_ and washing twice with water, once with brine and drying over Na_2_SO_4_. After removing the solvent under reduced pressure, the crude product was purified by flash chromatography and recrystallization.

#### **8,8'-[9-[2-(2-Ethoxyethoxy)ethyl]-9*H*-carbazole-2,7-diyl]bis[2-(2,6-diisopropylphenyl)-1*H*-benzo[10,5]anthra[2,1,9-*def*]isoquinoline-1,3(2*H*)-dione] (PMI-[C-OEG], C**_**86**_**H**_**71**_**N**_**3**_**O**_**6**_**)**

Synthesis was done by following the general Suzuki procedure. Used amounts: 960 mg compound **7** (2*.*18 mmol), 550 mg compound **5** (0*.*905 mmol), 10 cm^3^ 1 M K_2_CO_3_ (10 mmol), 2 drops Aliquat 336, 100 mg [Pd(PPh_3_)_4_] (0*.*080 mmol), 30 cm^3^ toluene. Reflux for 17 h. Red–black crude product (1*.*30 g). Column eluent was CH_2_Cl_2_:acetone 50:1, the recrystallization solvent was toluene. Yield 134 mg (12%); red-violet solid; *R*_*f*_ = 0.48 (CH_2_Cl_2_:acetone 60:1); glass transition: 276 °C; m.p.: 285 °C; ^1^H NMR (500 MHz, CDCl_3_): *δ* = 8.72–8.62 (m, 4H), 8.53 (d, ^3^*J*_HH_ = 7.8 Hz, 2H), 8.51–8.40 (m, 6H), 8.33 (d, ^3^*J*_HH_ = 7.8 Hz, 2H), 8.16 (d, ^3^*J*_HH_ = 8.4 Hz, 2H), 7.80–7.70 (m, 4H), 7.62 (t, ^3^*J*_HH_ = 7.8 Hz, 2H), 7.55–7.45 (m, 4H), 7.36 (d, ^3^*J*_HH_ = 7.8 Hz, 4H), 4.65 (t, ^3^*J*_HH_ = 5.1 Hz, 2H), 3.96 (t, ^3^*J*_HH_ = 5.6 Hz, 2H), 3.55 (t, ^3^*J*_HH_ = 4.5 Hz, 2H), 3.43 (t, ^3^*J*_HH_ = 4.5 Hz, 2H), 3.28 (q, ^3^*J*_HH_ = 7.0 Hz, 2H), 2.81 (sept, ^3^*J*_HH_ = 6.8 Hz, 4H), 1.21 (d, ^3^*J*_HH_ = 6.8 Hz, 24H), 0.97 (t, ^3^*J*_HH_ = 7.0 Hz, 3H) ppm; ^13^C NMR (125 MHz, CDCl_3_, ATP): *δ* = 164.0, 145.8, 144.3, 141.5, 137.9, 137.8, 137.7, 133.0, 132.1, 132.1, 131.1, 130.6, 129.8, 129.5, 129.4, 128.7, 128.5, 128.5, 127.1, 127.0, 124.0, 124.0, 123.5, 122.4, 121.9, 121.0, 120.9, 120.4, 120.3, 120.1, 110.8, 71.1, 69.9, 69.6, 66.7, 43.6, 29.2, 24.1, 15.0 ppm; HR-MS (MALDI-TOF): *m/z* calcd. C_86_H_72_N_3_O_6_^+^ ([M + H]^+^) 1242.5421, found 1242.5321; UV–Vis (CHCl_3_): *λ*_max_ (*α*) = 532 (9.2 × 10^4^), 507 nm (8.2 × 10^4^) nm (dm^3^ mol^−1^ cm^−1^); Fluorescence (CHCl_3_, excit. 485 nm): *λ*_max_ (*I*_rel_) = 593 (1).

#### **8,8'-[9,9-Bis[2-(2-ethoxyethoxy)ethyl]-9*H*-fluorene-2,7-diyl]bis[2-(2,6-diisopropylphenyl)-1*H*-benzo[10,5]anthra[2,1,9-*def*]isoquinoline-1,3(2*H*)-dione] (PMI-[F-OEG], C**_**93**_**H**_**84**_**N**_**2**_**O**_**8**_**)**

Synthesis was done by following the general Suzuki procedure. Used amounts: 340 mg compound **7** (0.62 mmol), 154 mg compound **6** (0.250 mmol), 2.80 cm^3^ 1 M K_2_CO_3_ (2.80 mmol), 1 spatula tip TBAB, 40.0 mg [Pd(PPh_3_)_4_] (0.035 mmol), 35 cm^3^ toluene. Reflux for 5 h. Red–black crude product. Column eluent was CH_2_Cl_2_:acetone 50:1, the recrystallization solvent was CH_2_Cl_2_ (solvent) and methanol (antisolvent). Yield 180 mg (51%); dark red solid; *R*_*f*_ = 0.20 (CH_2_Cl_2_:acetone 40:1), 0.05 (CH_2_Cl_2_:acetone 99:1); glass transition: 222 °C; m.p.: 345 °C; ^1^H NMR (500 MHz, CDCl_3_): *δ* = 8.70 (d, ^3^*J*_HH_ = 4.6 Hz, 2H), 8.68 (d, ^3^*J*_HH_ = 4.6 Hz, 2H), 8.60–8.46 (m, 8H), 8.11 (d, ^3^*J*_HH_ = 8.4 Hz, 2H), 7.97 (d, ^3^*J*_HH_ = 7.7 Hz, 2H), 7.72 (d, ^3^*J*_HH_ = 7.7 Hz, 2H), 7.69–7.58 (m, 6H), 7.49 (t, ^3^*J*_HH_ = 7.7 Hz, 2H), 7.36 (d, ^3^*J*_HH_ = 7.8 Hz, 4H), 3.45–3.38 (m, 8H), 3.38–3.32 (m, 4H), 3.11 (t, ^3^*J*_HH_ = 7.1 Hz, 4H), 2.80 (sept, ^3^*J*_HH_ = 6.8 Hz, 4H), 2.54 (t, ^3^*J*_HH_ = 6.9 Hz, 4H), 1.20 (d, ^3^*J*_HH_ = 6.8 Hz, 24H), 1.12 (t, ^3^*J*_HH_ = 7.0 Hz, 6H) ppm; ^13^C NMR (125 MHz, CDCl_3_): *δ* = 164.0, 150.0, 145.8, 143.5, 139.9, 139.3, 137.7, 137.6, 132.8, 132.1, 132.1, 131.1, 130.6, 129.64, 129.53, 129.47, 129.32, 128.66, 128.53, 128.46, 128.24, 127.2, 127.0, 125.0, 124.0, 123.6, 121.04, 120.94, 120.40, 120.24, 120.17, 70.2, 69.8, 67.5, 66.6, 51.8, 39.6, 29.2, 24.1, 15.1 ppm; HR-MS (MALDI-TOF): *m/z* calcd. C_93_H_84_N_2_O_8_^+^ (M^+^) 1356.6228, found 1356.6490; UV–Vis (CHCl_3_): *λ*_max_ (*α*) = 530 (9.2 × 10^4^), 507 (8.3 × 10^4^) nm (dm^3^ mol^−1^ cm^−1^); Fluorescence (CHCl_3_, excit. 485 nm): *λ*_max_ (*I*_rel_) = 585 nm (1); FT-IR: $\overline{V}$  = 1701, 1661 (imide) cm^−1^.

### Supplementary Information

Below is the link to the electronic supplementary material.

Supplementary file1 (PDF 4483 KB)
