# Supplementary material for: Glycol bearing perylene monoimide based non-fullerene acceptors with increased dielectric permittivity
Source: Monatsh Chem. 2022 Jul 29;154(12):1369–81. doi: 10.1007/s00706-022-02956-2 (PMC10667137; doi:10.1007/s00706-022-02956-2)
Supplement: Supplementary file 1 — Supplementary file1 (PDF 4483 KB) [file 706_2022_2956_MOESM1_ESM.pdf]

Supplementary Information

Glycol bearing perylene monoimide based non-fullerene acceptors with increased dielectric permittivity

Peter Fürk[1] ● Jakob Hofinger[2] ● Matiss Reinfelds[1] ● Thomas Rath[1] ● Heinz Amenitsch[3] ● Markus Clark Scharber[2] ● Gregor Trimmel[1]

✉ Gregor Trimmel
gregor.trimmel@tugraz.at

1 Institute for Chemistry and Technology of Materials (ICTM), NAWI Graz, Graz University of Technology, Stremayrgasse 9, 8010 Graz, Austria

2 Linz Institute for Organic Solar Cells (LIOS), Institute of Physical Chemistry, Johannes Kepler University Linz, Altenbergerstrasse 69, 4040 Linz, Austria

3 Institute of Inorganic Chemistry, NAWI Graz, Graz University of Technology, Stremayrgasse 9, 8010 Graz, Austria

Content

Synthesis 2
NMR spectra 6
Mass spectra 11
IR spectra 13
Computations 14
Thermoanalysis 17
Dielectric properties 19
Photovoltaic Properties 20
References 20

Synthesis

1-Bromo-2-(2-ethoxyethoxy)ethane **(2)**

2-(2-Ethoxyethoxy)ethanol (**1**) (9.65 g, 71.9 mmol) was dissolved in 40 ml CH_2Cl_2 in a round bottom flask equipped with a stir bar and septum. Then, freshly distilled PBr_3 (2.70 ml, 28.7 mmol) was added dropwise over 1 h at 0 °C. The mixture was then stirred at RT for 26 h. Workup was done by diluting the reaction mixture with 40 ml CH_2Cl_2 and washing it with 3 × 20 ml water. The solvent was evaporated and the crude product, an opaque brown liquid, was purified by fractional distillation in nitrogen atmosphere (product fraction at 140-170 °C). Yield 6.94 g (69 %), light brown liquid, R_f 0.84 (cyclohexane:acetone 1:1). - ^{1}H NMR (300 MHz, $CDCl_3$, TMS) δ (ppm): 3.81 (t, $^3J_{HH}$ = 6.5 Hz, 2H), 3.72 - 3.53 (m, 2H), 3.63 - 3.42 (m, 6H), 1.22 (t, $^3J_{HH}$ = 7.0 Hz, 3H). - ^{13}C NMR (75 MHz, $CDCl_3$, TMS) δ (ppm): 71.90, 70.3, 69.6, 66.4, 30.1, 14.9. The ^{1}H and ^{13}C NMR spectra were found to be identical to literature [1].

2,7-dibromo-9,9-bis(2-(2-ethoxyethoxy)ethyl)-9H-fluorene **(6)**

2,7-dibromofluorene (**4**) (1.41 g, 4.33 mmol), tetrabutyl ammoniumbromide (69 mg, 0.21 mmol) and NaOH (15.0 g, 375 mmol) were weighed in a 3-neck round bottom flask with a stir bar, reflux condenser and nitrogen inlet. 50 ml toluene and 25 ml water were added and the flask was flushed for 5 min with nitrogen. The mixture was subsequently stirred at 80 °C for 23 h. The organic phase turned dark red-violet. For workup, the reaction mixture was diluted with 150 ml CH_2Cl_2, washed with 2 × 100 ml water and 50 ml brine (organic phase turned red-green) and dried over Na_2SO_4 (organic phase turned green). Filtration and evaporation of the solvent under reduced pressure gave both green and colourless crystals. The crude product was purified by flash chromatography (gradient petrol ether : ethyl acetate 8:1 to 1:1) to give colourless crystals.

Yield 1.50 mg (63 %), colourless solid, R_f 0.35 (petrol ether : ethyl acetate 5:1). - ^{1}H NMR (300 MHz, $CDCl_3$, TMS) δ (ppm): 7.60-7.40 (m, 6H), 3.42 (q, $^3J_{HH}$ = 6.8 Hz, 4H), 3.34 (t, $^3J_{HH}$ = 4.3 Hz, 4H), 3.20 (t, $^3J_{HH}$ = 4.3 Hz, 4H), 2.97 (t, $^3J_{HH}$ = 7.2 Hz, 4H), 2.35 (t, $^3J_{HH}$ = 7.2 Hz, 4H), 1.15 (t, $^3J_{HH}$ = 6.9 Hz, 6H). - ^{13}C NMR (75 MHz, $CDCl_3$, TMS) δ (ppm): 151.0, 138.5, 130.8, 126.8, 121.8, 121.3, 70.2, 69.8, 66.9, 66.7, 52.0, 39.6, 15.2. The ^{1}H NMR spectrum found to be identical to literature [2].

2-(2,6-Diisopropylphenyl)-1H-benzo[10,5]anthra[2,1,9-def]isoquinoline-1,3(2H)-dione (**7a**)

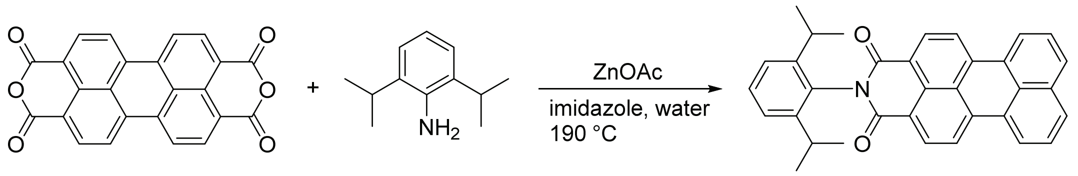

In an autoclave, perylene-3,4,9,10-tetracarboxylic dianhydride (4.0 g, 10.2 mmol, 1 equiv.), zinc acetate (1.51 g, 6.86 mmol, 0.67 equiv.) and imidazole (20.4 g) were dispersed in 4.8 ml water. 2,6-diisopropylaniline (1.014 ml, 5.38 mmol, 0.53 equiv.) was added and the closed crucible was placed in a muffle furnace at 190 °C for 24 h. Afterwards, the reaction mixture was cooled to RT, rinsed with water, acidified to pH 2 and filtered. The filter cake was extracted with $CHCl_3$ with a Soxhlet extractor for approx. 48 h. The crude product was then purified by column chromatography with $CHCl_3$ as eluent to give the product as a red solid. Yield: 1.03 g (40 %). R_f = 0.30-0.36 ($CHCl_3$). - ^{1}H NMR (300 MHz, $CDCl_3$) δ (ppm): 8.61 (d, J = 8.1 Hz, 2H), 8.40-8.35 (m, 4H), 7.87 (d, J = 8.1 Hz, 2H), 7.60 (t, J = 7.8 Hz, 2H), 7.48 (t, J = 7.6 Hz, 1H), 7.35 (d, J = 7.6 Hz, 2H), 2.78 (sept, J = 6.9 Hz, 2H), 1.20 (d, J = 6.9 Hz, 12H). - ^{13}C NMR (75 MHz, $CDCl_3$) δ (ppm): 164.1, 145.8, 137.6, 134.4, 132.1, 131.2, 131.1, 130.7, 129.6, 129.3, 128.1, 127.2, 124.2, 123.9, 121.1, 120.3, 29.3, 24.2. The ^{1}H and ^{13}C spectra were found to be identical to literature [3, 4].

8-Bromo-2-(2,6-iisopropylphenyl)-1H-benzo[10,5]anthra[2,1,9-def]-isoquinoline-1,3(2H)-dione (**7b**)

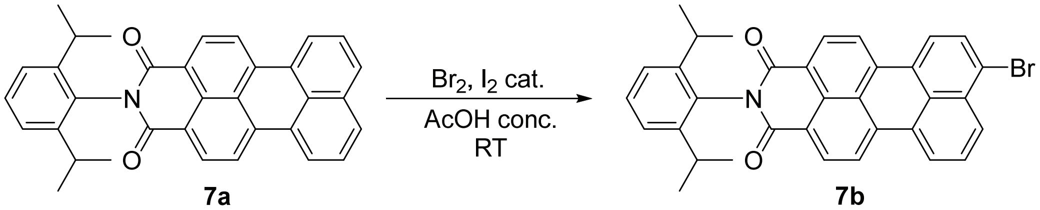

7a **7b**

Compound **7a** (6.00 g, 12.5 mmol) was suspended in 255 ml glacial acetic acid in a 500 ml round bottom flask and a heavy stir bar. After 30 min stirring, I_2 (125 mg, 0.50 mmol) was added. Then, Br_2 (2.50 ml, 50.0 mmol) was added over 15 min. Stirring at RT for 24 h. When TLC indicated reaction completion, the excess Br_2 was removed by bubbling air through the solution for 1 h. 150 ml methanol was added and after 30 min the mixture was poured onto 1 l water. The product was isolated by filtration and washing the residue with water until the filtrate had a pH value of ~ 6 (controlled with indicator paper). The residue was dried over KOH pellets and $CaCl_2$.

Yield 6.61 g (94 %), dark red solid, R_f = 0.60 (toluene:acetone 99:1, eluted 5 times). - ^{1}H NMR (300 MHz, $CDCl_3$, TMS) δ (ppm): 8.71 - 8.59 (m, 2H), 8.51 - 8.42 (m, 2H), 8.40 (d, $^3J_{HH}$ = 8.1 Hz, 1H), 8.30 (d, $^3J_{HH}$ = 8.2 Hz, 1H), 8.22 (d, $^3J_{HH}$ = 8.2 Hz, 1H), 7.90 (d, $^3J_{HH}$ = 8.2 Hz, 1H), 7.71 (t, $^3J_{HH}$ = 8.1 Hz, 1H), 7.49 (t, $^3J_{HH}$ = 7.6 Hz, 1H), 7.34 (d, $^3J_{HH}$ = 7.7 Hz, 2H), 2.77 (sept, $^3J_{HH}$ = 6.7 Hz, 2H), 1.19 (d, $^3J_{HH}$ = 6.8 Hz, 12H). - FT-IR $\tilde{\nu}$ (cm^{-1}): 1697, 1653 (OCNCO imide). The ^{1}H and ^{13}C spectra were found to be identical to literature [3, 4].

2-(2,6-Diisopropylphenyl)-8-(4,4,5,5-tetramethyl-1,3,2-dioxaborolan-2-yl)-1H-benzo[10,5]anthra[2,1,9-def]isoquinoline-1,3(2H)-dione (**7**)

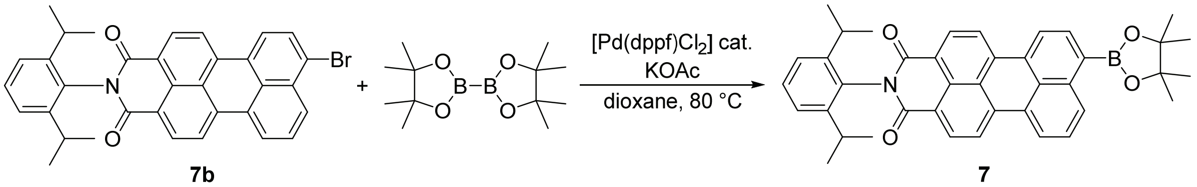

7b **7**

The reaction was done under exclusion of air and water in nitrogen atmosphere. All starting materials and reactants were dried beforehand. A flame-dried 3-neck round bottom flask equipped with a reflux condenser and nitrogen inlet was charged with compound **7b** (1.21 g, 2.16 mmol), bis(pinacolato)diboron (724 mg, 2.79 mmol), KOAc (850 mg, 7.58 mmol), [1,1'-bis(diphenylphosphino)-ferrocene]dichloropalladium(II) ([Pd(dppf)Cl_2], 328 mg, 0.45 mmol) and 50 ml dioxane anh. The mixture was stirred at 80 °C for 24 h. Reaction progress was monitored by TLC. For workup, the reaction mixture was diluted with 200 ml CH_2Cl_2 and washed with 3 × 50 ml water, brine and dried over Na_2SO_4. Purification was done by flash chromatography (gradient CH_2Cl_2:CH 5:1 to pure CH_2Cl_2).

Yield 800 mg (59 %), red solid, R_f = 0.85 (CH_2Cl_2). - ^{1}H NMR (300 MHz, $CDCl_3$, TMS) δ (ppm): 8.85 (d, $^3J_{HH}$ = 8.4 Hz, 1H), 8.63 (t, $^3J_{HH}$ = 7.0 Hz, 2H), 8.50-8.31 (m, 4H), 8.19 (d, $^3J_{HH}$ = 7.4 Hz, 1H), 7.64 (dd, $^3J_{HH}$ = 7.8 Hz, 1H), 7.48 (dd, $^3J_{HH}$ = 7.6 Hz, 1H), 7.35 (d, $^3J_{HH}$ = 7.6 Hz, 2H), 2.78 (dq, $^3J_{HH}$ = 6.7 Hz, 2H), 1.47 (s, 12H), 1.19 (d, $^3J_{HH}$ = 6.6 Hz, 12H). - ^{13}C NMR (75 MHz, $CDCl_3$, TMS) δ (ppm): 164.2, 164.1, 145.9, 138.2, 138.0, 137.5, 137.4, 136.4, 132.2, 132.0, 131.9, 131.8, 131.2, 130.5, 129.6, 129.1, 127.9, 127.3, 127.0, 125.1, 124.1, 123.8, 122.9, 121.5, 120.9, 120.4, 84.4, 29.3, 25.1, 24.2. - HR-MS (MALDI-TOF) calcd. $C_{40}H_{39}NO_4B^+$ $[MH]^+$: 608.2979; found 608.4222. - FT-IR $\tilde{\nu}$ (cm^{-1}): 1699, 1661 (OCNCO imide). The ^{1}H spectrum was found to be identical to literature [3].

NMR spectra

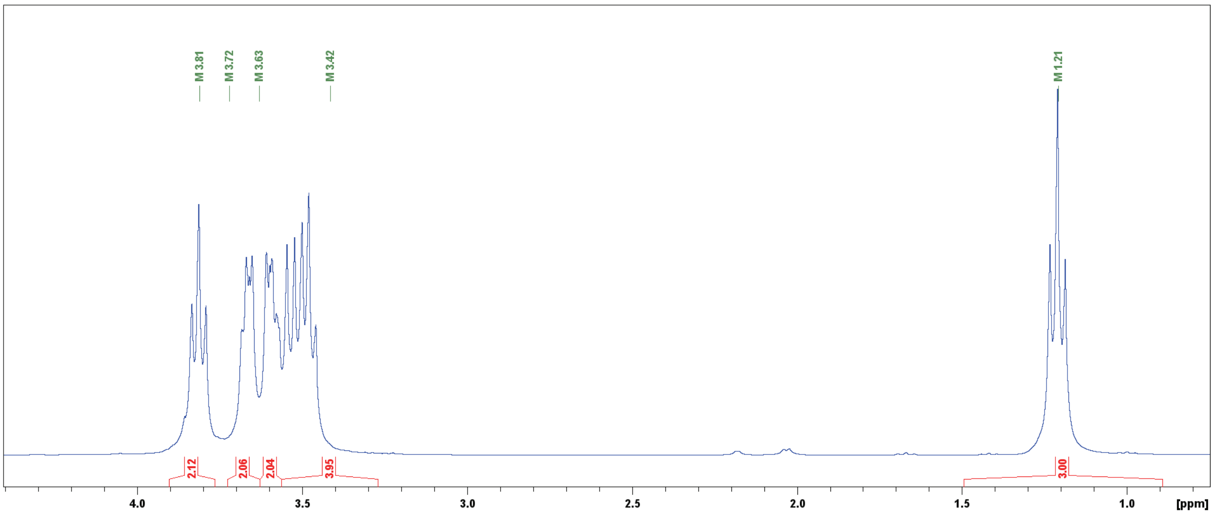

Fig. S1 ^{1}H NMR spectrum of compound **2** (300 MHz, $CDCl_3$, TMS)

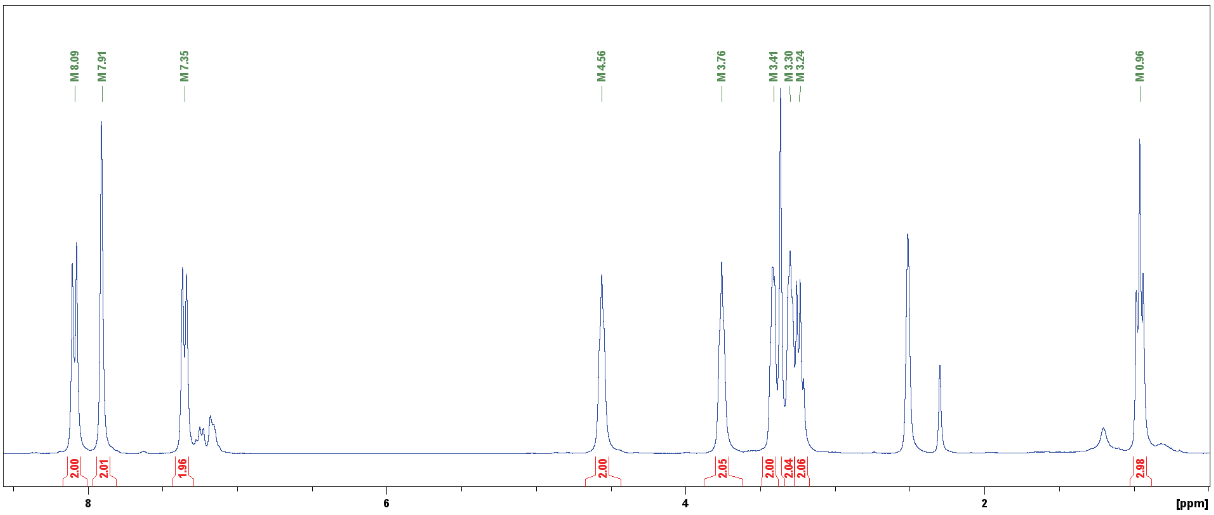

Fig. S2 ^{1}H NMR spectrum of compound **5** (300 MHz, $(CD_3)_2SO$, TMS)

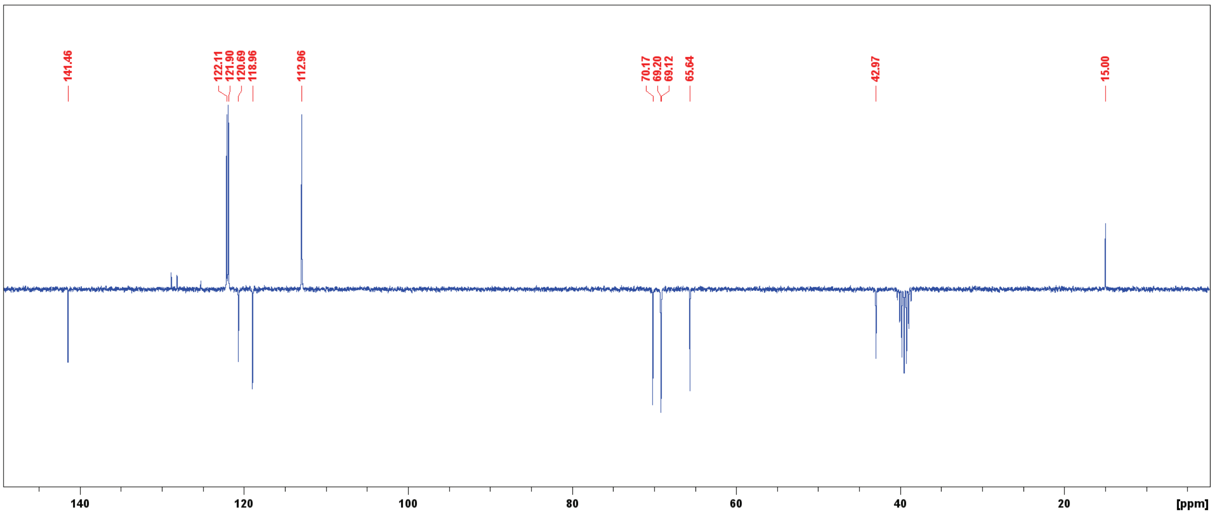

Fig. S3 APT-^{13}C NMR spectrum of compound **5** (75 MHz, $(CD_3)_2SO$, referenced to solvent signal)

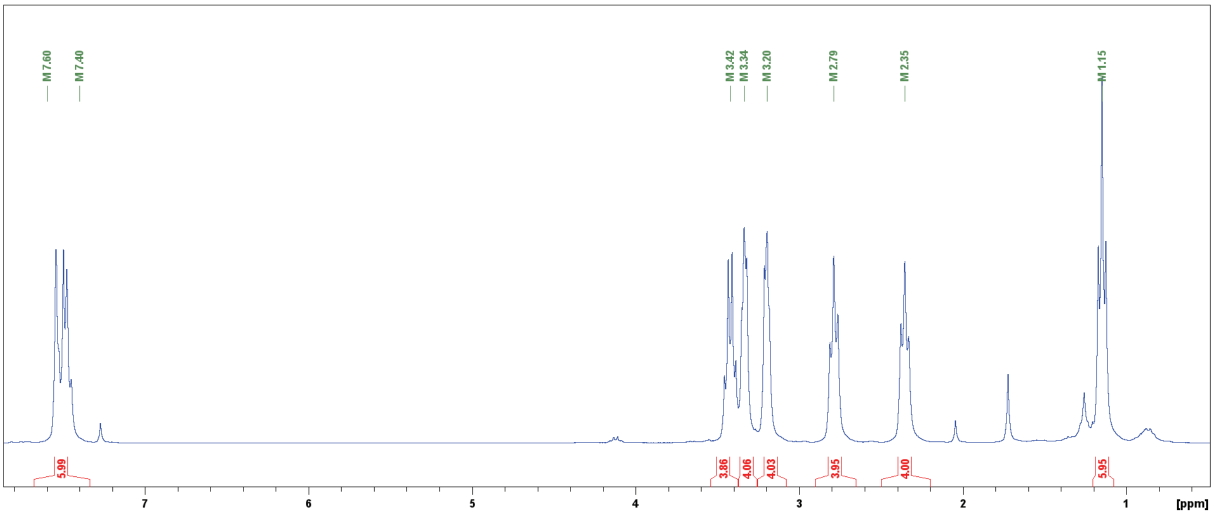

Fig. S4 ^{1}H NMR spectrum of compound **6** (300 MHz, $CDCl_3$, TMS)

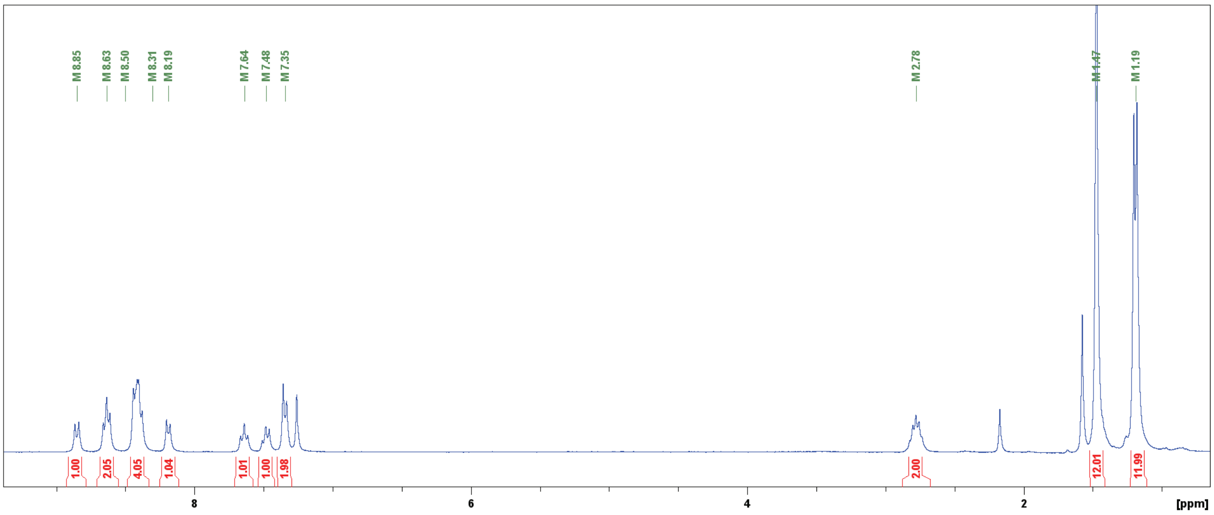

Fig. S5 ^{1}H NMR spectrum of compound **7** (300 MHz, $CDCl_3$, TMS)

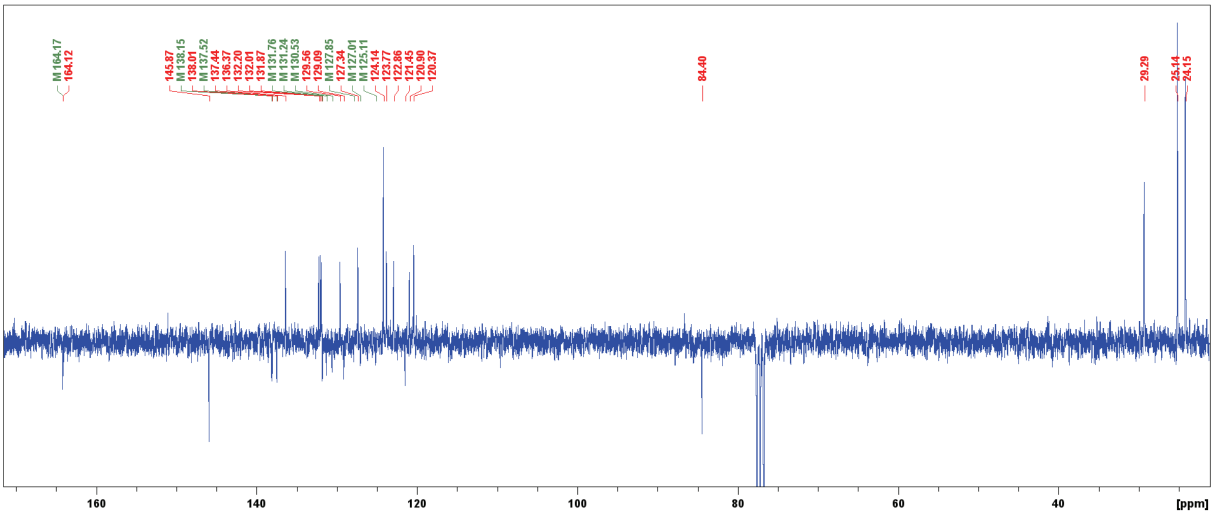

Fig. S6 APT-^{13}C NMR spectrum of compound **7** (125 MHz, $CDCl_3$, referenced to solvent signal)

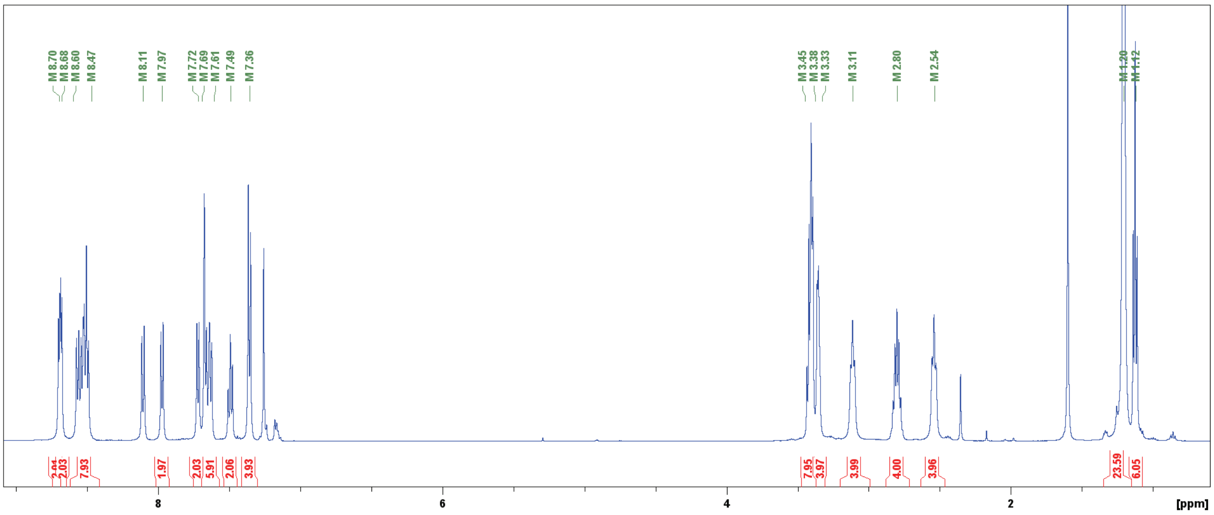

Fig. S7 ^{1}H NMR spectrum of compound PMI-[**F-OEG**] (500 MHz, $CDCl_3$, TMS)

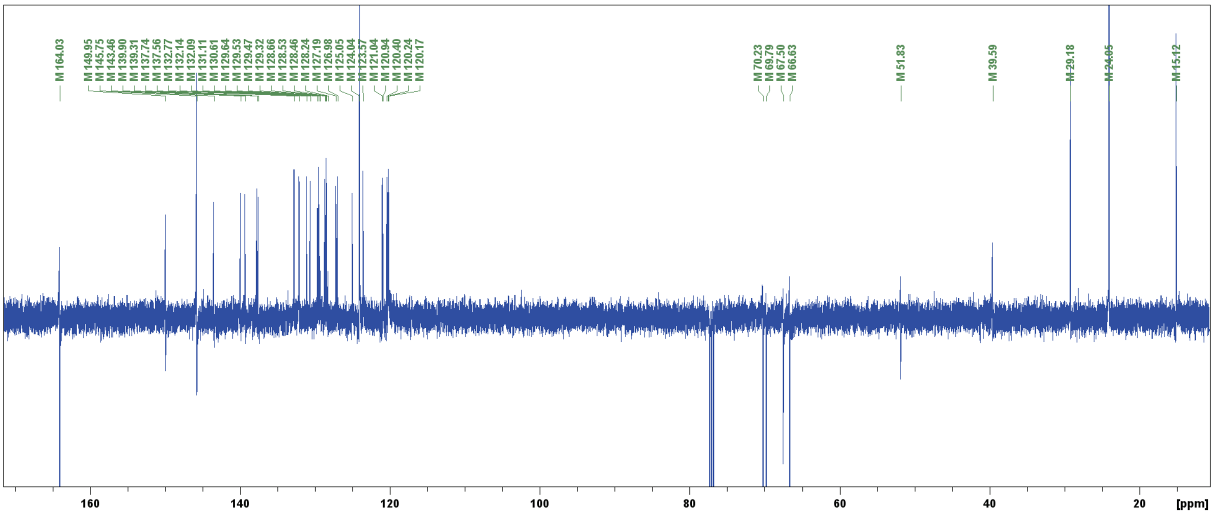

Fig. S8 APT-^{13}C NMR spectrum of compound PMI-[**F-OEG**] (125 MHz, $CDCl_3$, referenced to solvent signal)

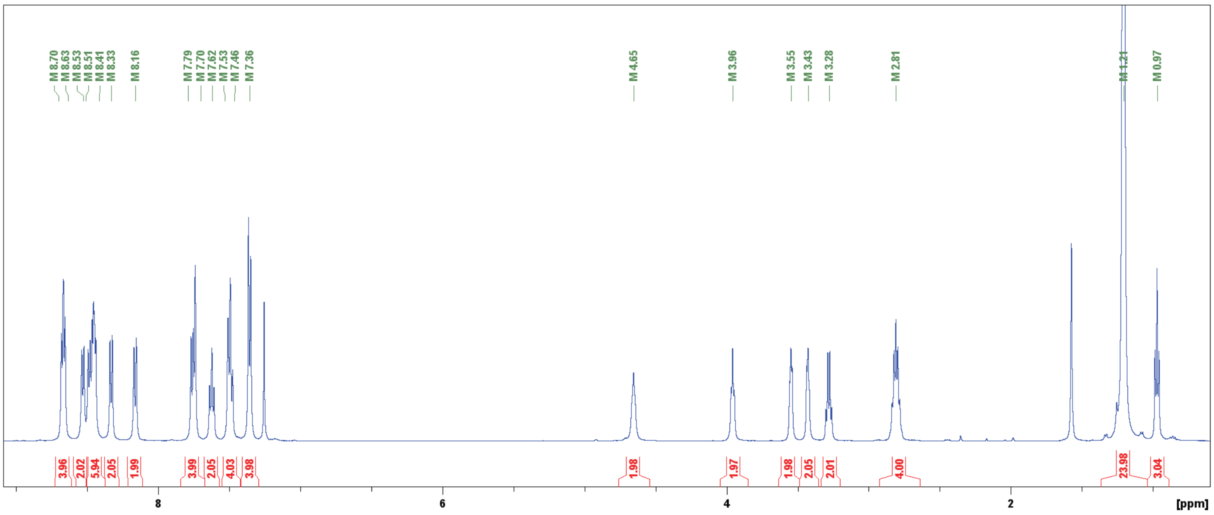

Fig. S9 ^{1}H NMR spectrum of compound PMI-[**C-OEG**] (500 MHz, $CDCl_3$, TMS)

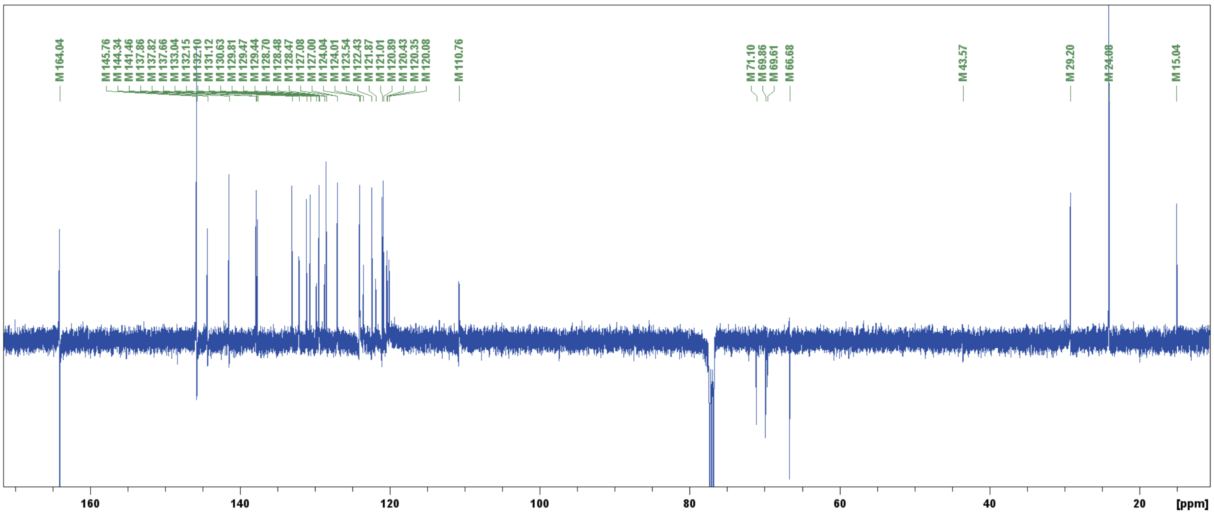

Fig. S10 APT-^{13}C NMR spectrum of compound PMI-[**C-OEG**] (125 MHz, $CDCl_3$, referenced to solvent signal)

Mass spectra

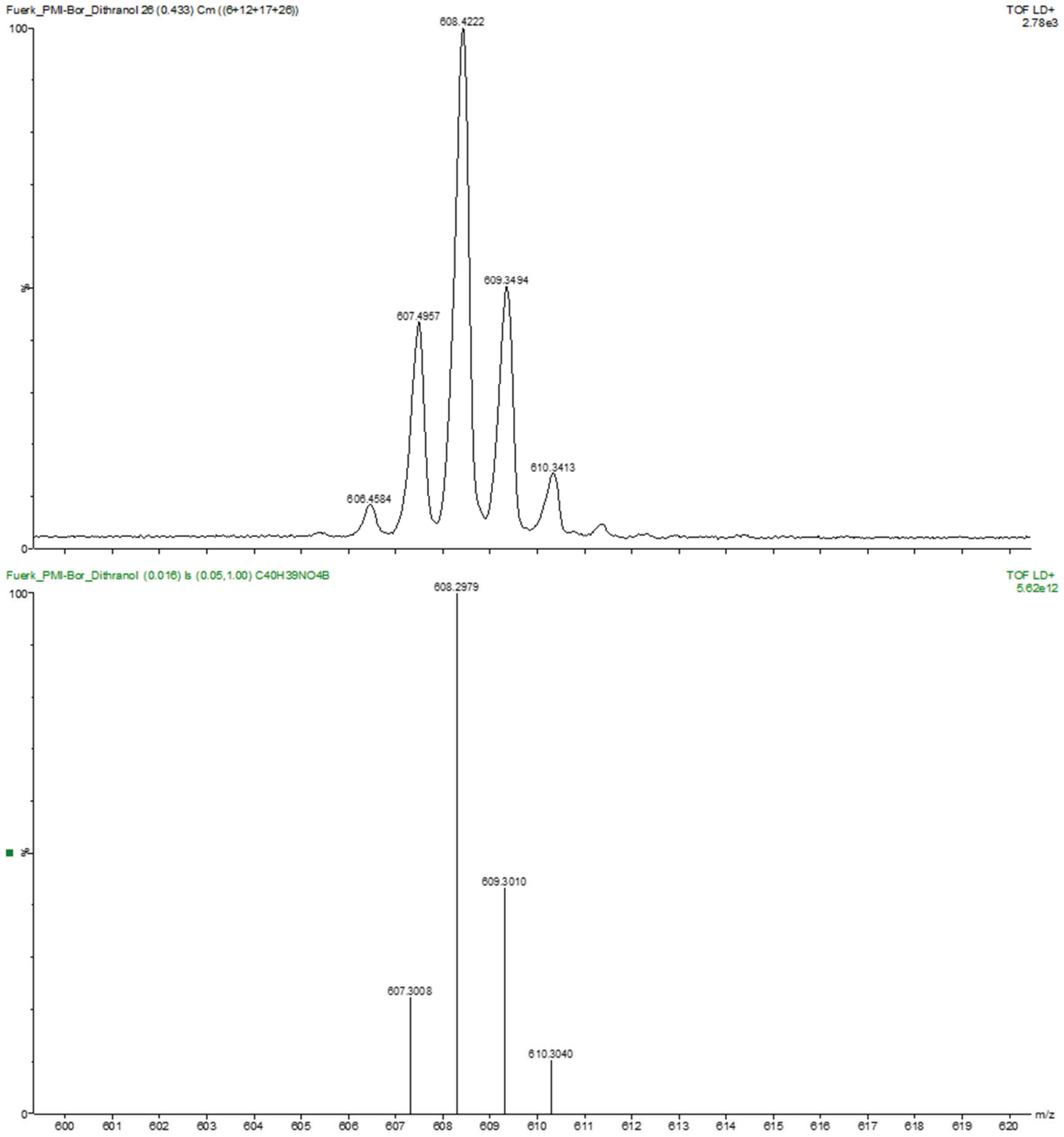

Fig. S11 MALDI-TOF HR-MS spectrum of compound **7** (matrix dithranol): experimental (**top**) and calculated (**bottom**) isotope pattern

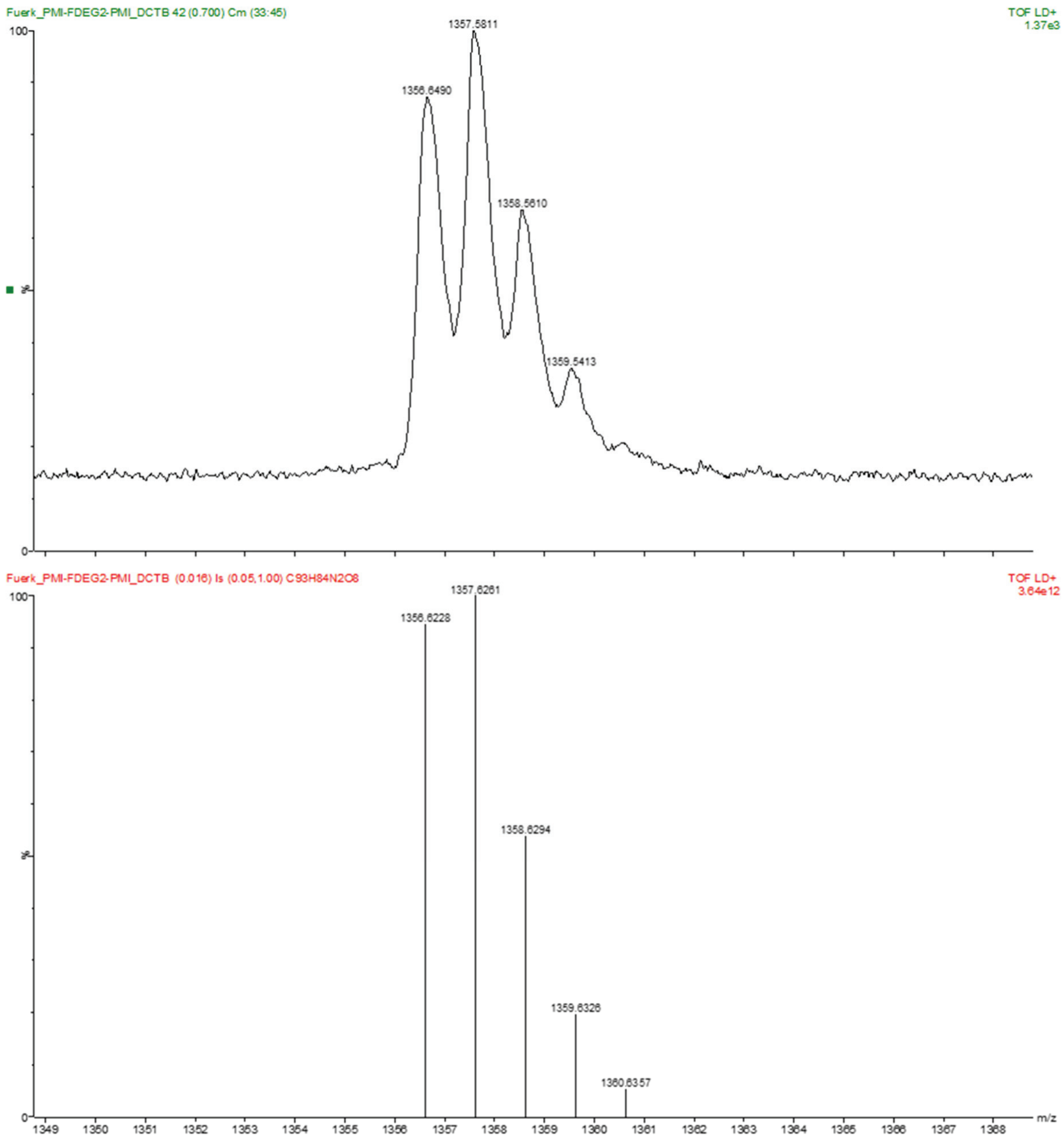

Fig. S12 MALDI-TOF HR-MS spectrum of compound PMI-[**F-OEG**] (matrix DCTB): experimental (**top**) and calculated (**bottom**) isotope pattern

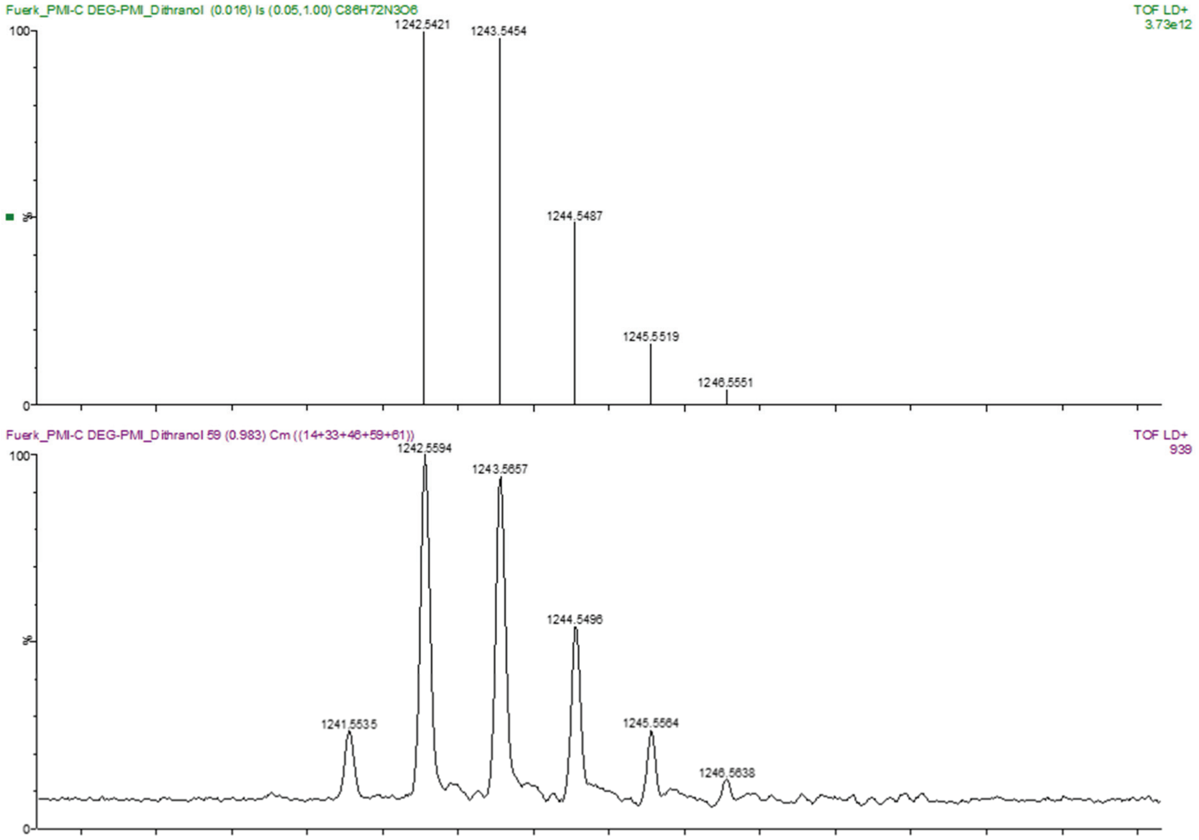

Fig. S13 MALDI-TOF HR-MS spectrum of compound PMI-[**C-OEG**] (matrix dithranol): experimental (**top**) and calculated (**bottom**) isotope pattern

IR spectra

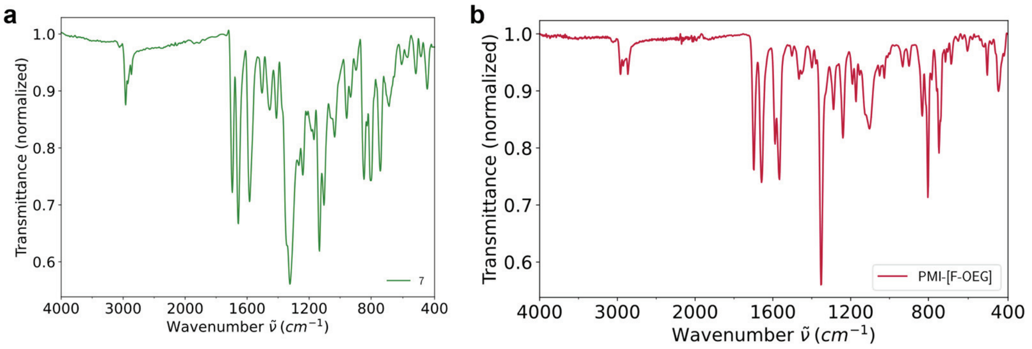

Fig. S14 FT-IR Spectra of (**a**) **7** and (**b**) PMI-[**F-OEG**]

Computations

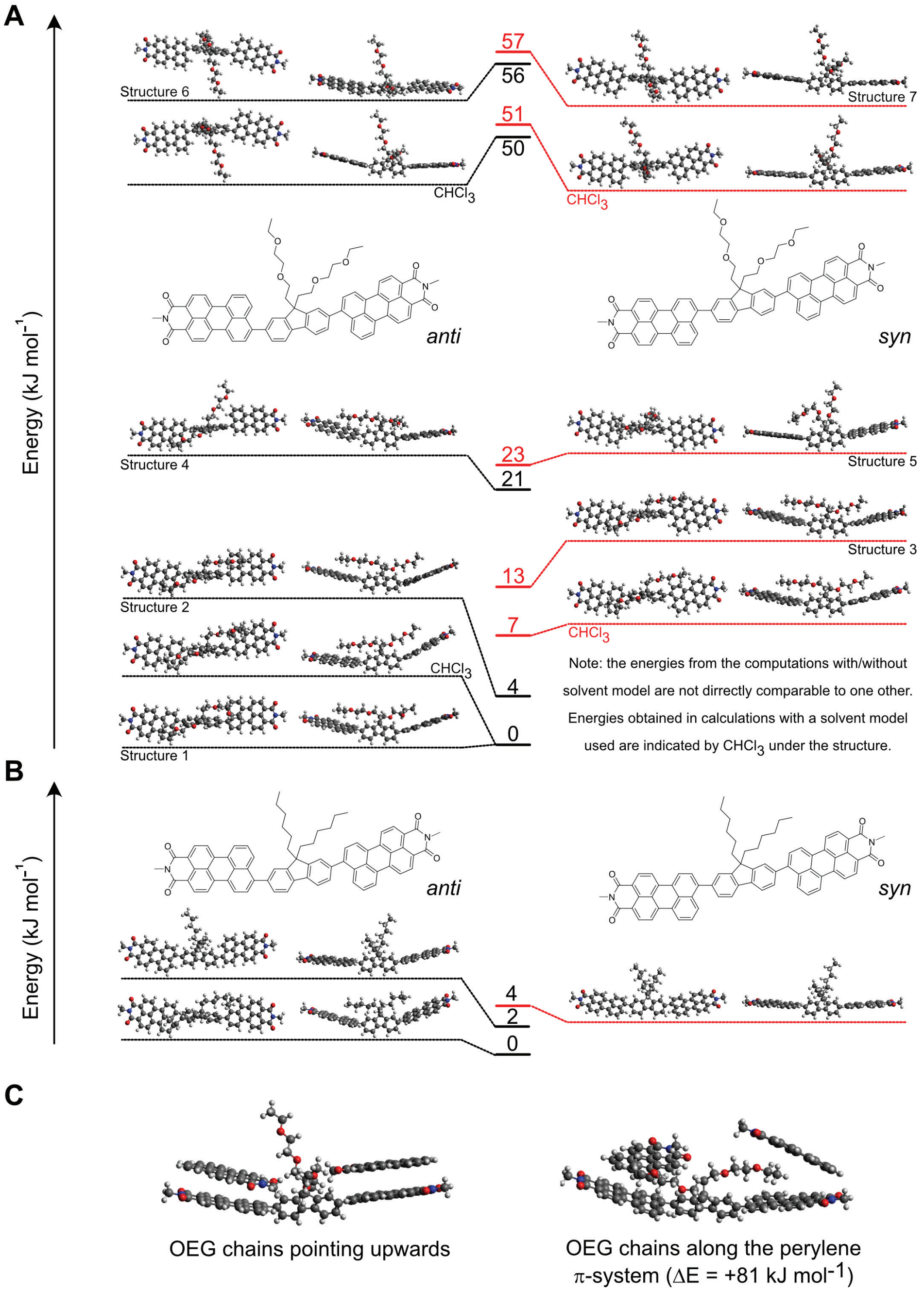

Fig. S15 (**a**) Energy for PMI-[**F-OEG**] with differently oriented OEG side chains. Syn isomer on the right side, anti isomer on the left side (red and black lines, respectively). (**b**) Energy for PMI-[**F-ALK**] with hexyl side chains oriented along or away from the perylene core. Syn isomer on the right side, anti isomer on the left side (red and black lines, respectively). Calculations of the structures shown in the **A** and **B** part of the figure are done on a B3LYP-GD3 level of theory using 6-31G(d,p) basis set. (**c**) Triads of PMI-[**F-OEG**] and two perylene monoimides (HF-3c level of theory).

In order to investigate how the molecular energy is influenced by the orientation of the oligoethylene glycol (OEG) side chains, we performed geometry optimization from various starting geometries. For each optimized geometry, we also performed frequency calculations to confirm that a local minimum is found (no negative frequencies). The lowest energy is achieved if the OEG side chains are located along the perylene core (**Fig. S15a**, structure 1). Small changes in the torsion angles in the side chains results in minimal increase of the energy as shown by the structure 2 [5]. Changing the orientation of the perylenes to syn also increases the energy (structure 3), however, a larger increase in energy happens (ΔE = 21 – 23 kJ mol^{-1}) if one of the OEG chains is pointing away from the perylene core (structures 4 and 5). If both side chains are pointing away from the perylene core, the energy is the highest (structures 6 and 7, ΔE = 56 – 57 kJ mol^{-1}). In reference compound with two hexyl chains (**Fig. S15b**) the energy difference between the two side chain orientations is only 2 kJ mol^{-1} (favoring orientation along the perylene core).

Performing geometry optimization of the same input geometry, but with a polarizable continuum model ($CHCl_3$ as solvent, see the $CHCl_3$ label under the structures in **Fig. S15a**), does not change the overall trend. However, the structures in which the side chains are pointing away from the perylene core are slightly less energetically higher (ΔE = 50 – 51 kJ mol^{-1}).

Nevertheless, in solid state the optimal orientation of the side chains might be different than for the isolated molecule. Especially, since a strong intermolecular π - π interaction between perylene moieties might be energetically desirable and the side chains would disturb that. We probed this by performing a geometry optimization of PMI-[**F-OEG**] molecule with two isolated perylene monoimides stacked on top. Doing such calculation with DFT methods would be expensive, thus we used HF-3c method which is computationally much faster. These

computations were done using Orca 4.2. It was found that a structure in which the OEG side chains are pointing upwards is energetically more favorable (ΔE = 81 kJ mol^{-1}).

Table S1 Calculated excitation properties of PMI-[**F-OEG**] and PMI-[**C-OEG**]. Calculations were done on a B3LYP-GD3 level of theory using 6-31G+(d,p) basis set.

Compound	State	Orbitals[a]	Coefficients	eV	nm	Oscillator strengths
PMI-[**F-OEG**] **isomer 1**	S_1	H-1→L	-0.11	2.21	562	0.85
		H→L	0.68			
		H→L+1	0.14			
	S_2	H-1→L	-0.29	2.33	533	0.53
		H→L	-0.18			
		H→L+1	0.61			
	S_3	H-1→L	0.63	2.40	517	0.15
		H→L+1	0.31			
	S_4	H-1→L+1	0.70	2.49	497	0.20
PMI-[**F-OEG**] **isomer 2**	S_1	H→L	0.70	2.26	548	1.34
	S_4	H-1→L+1	0.70	2.47	501	0.47
PMI-[**C-OEG**]	S_1	H→L	0.70	2.25	550	1.32
	S_4	H-1→L+1	0.69	2.47	502	0.46

[a] H stands for HOMO, L stands for LUMO.

Thermoanalysis

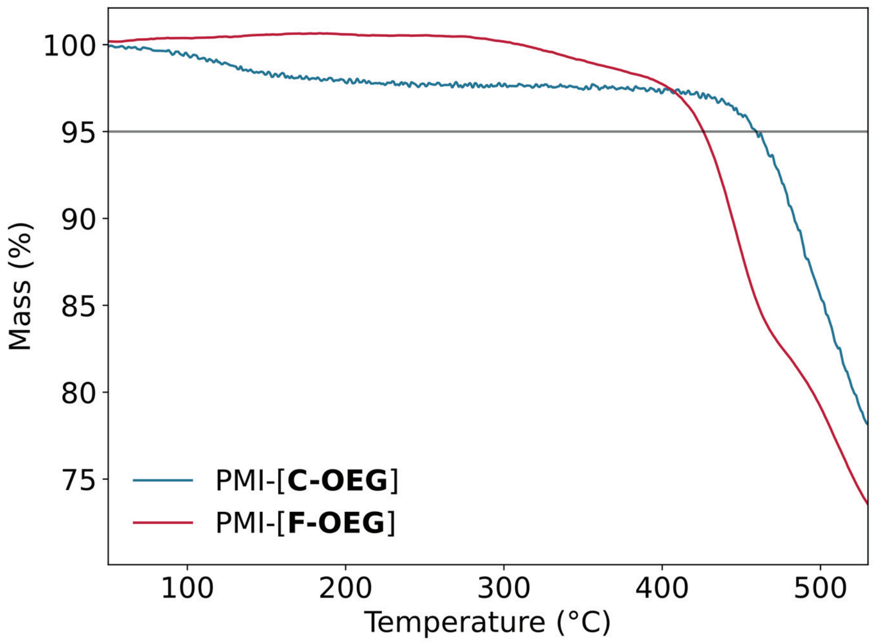

Fig. S16 Thermogravimetric analysis of both pristine acceptors

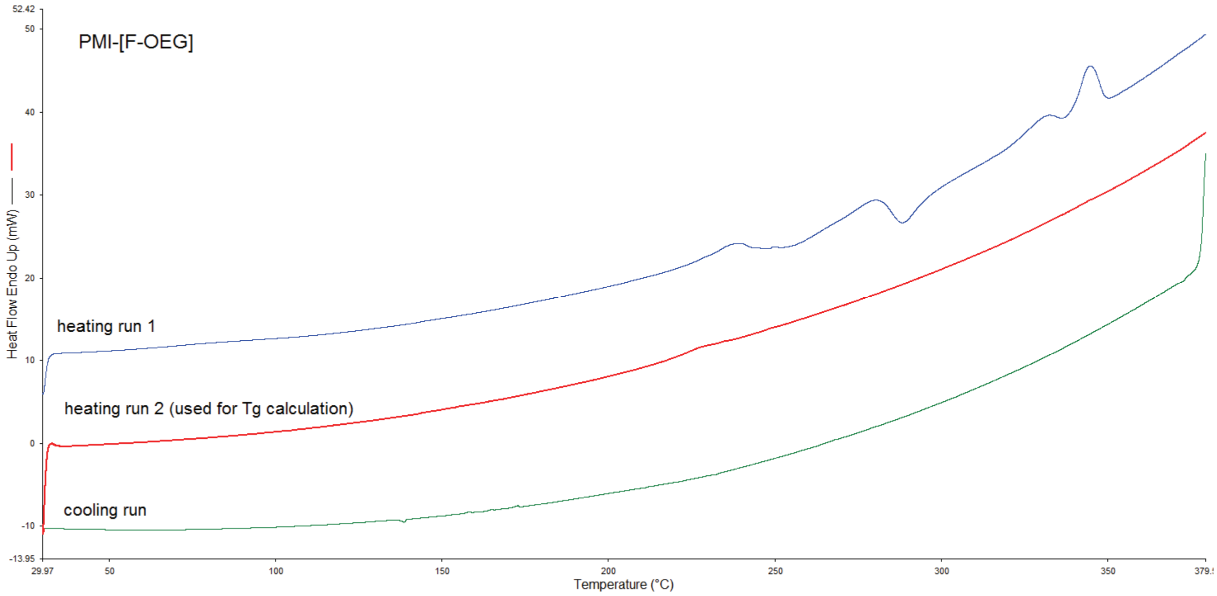

Fig. S17 DSC measurement of PMI-[**F-OEG**]

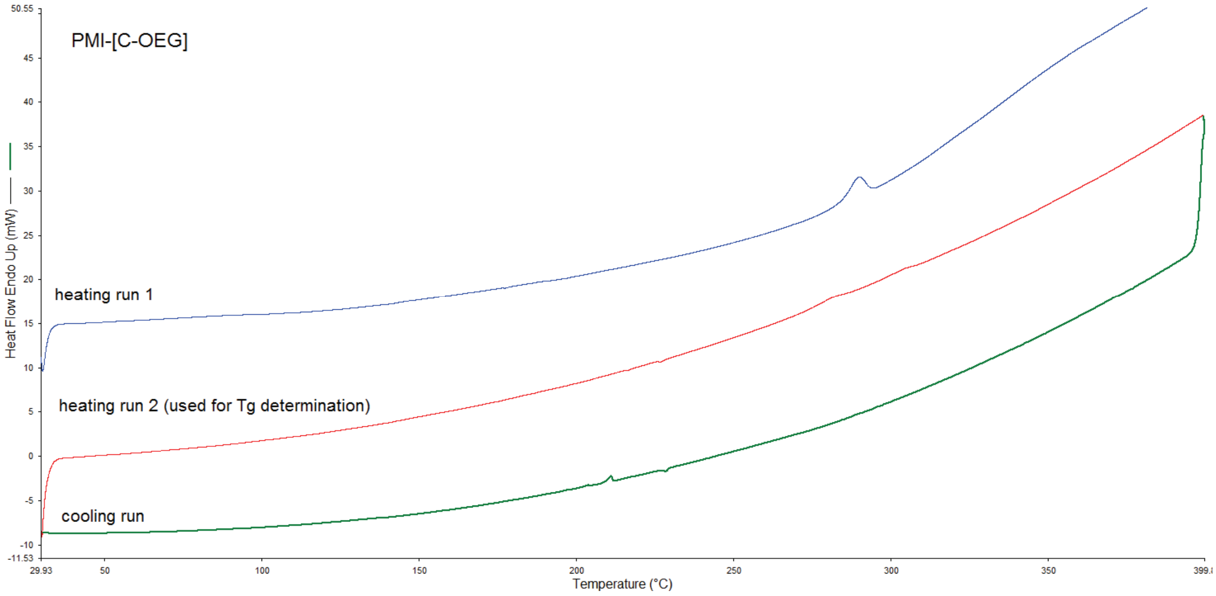

Fig. S18 DSC measurement of PMI-[**C-OEG**]

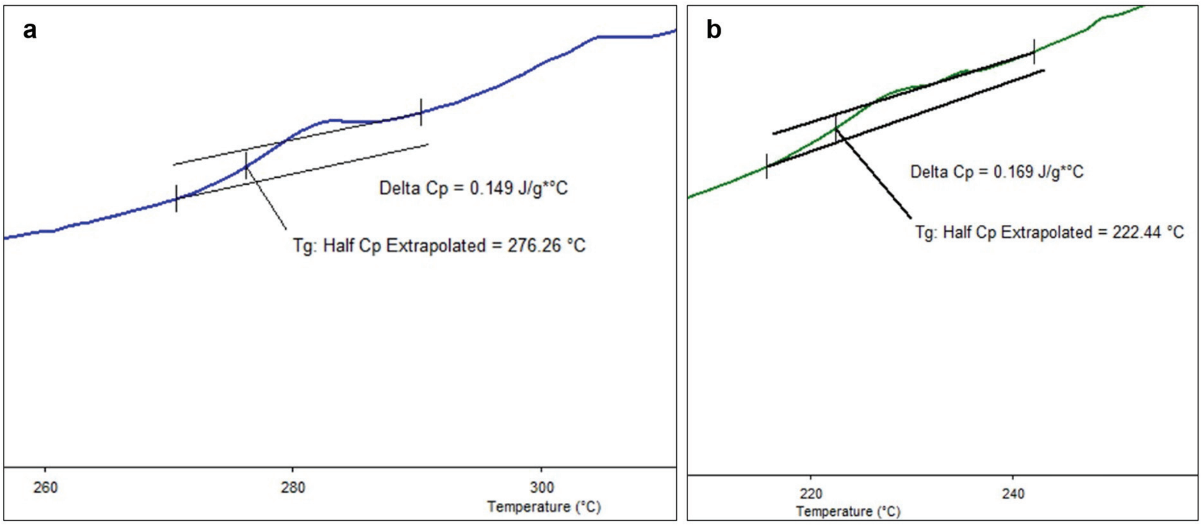

Fig. S19 DSC measurement: Close-up on glass transition of (**a**) PMI-[**C-OEG**] and (**b**) PMI-[**F-OEG**]

Dielectric properties

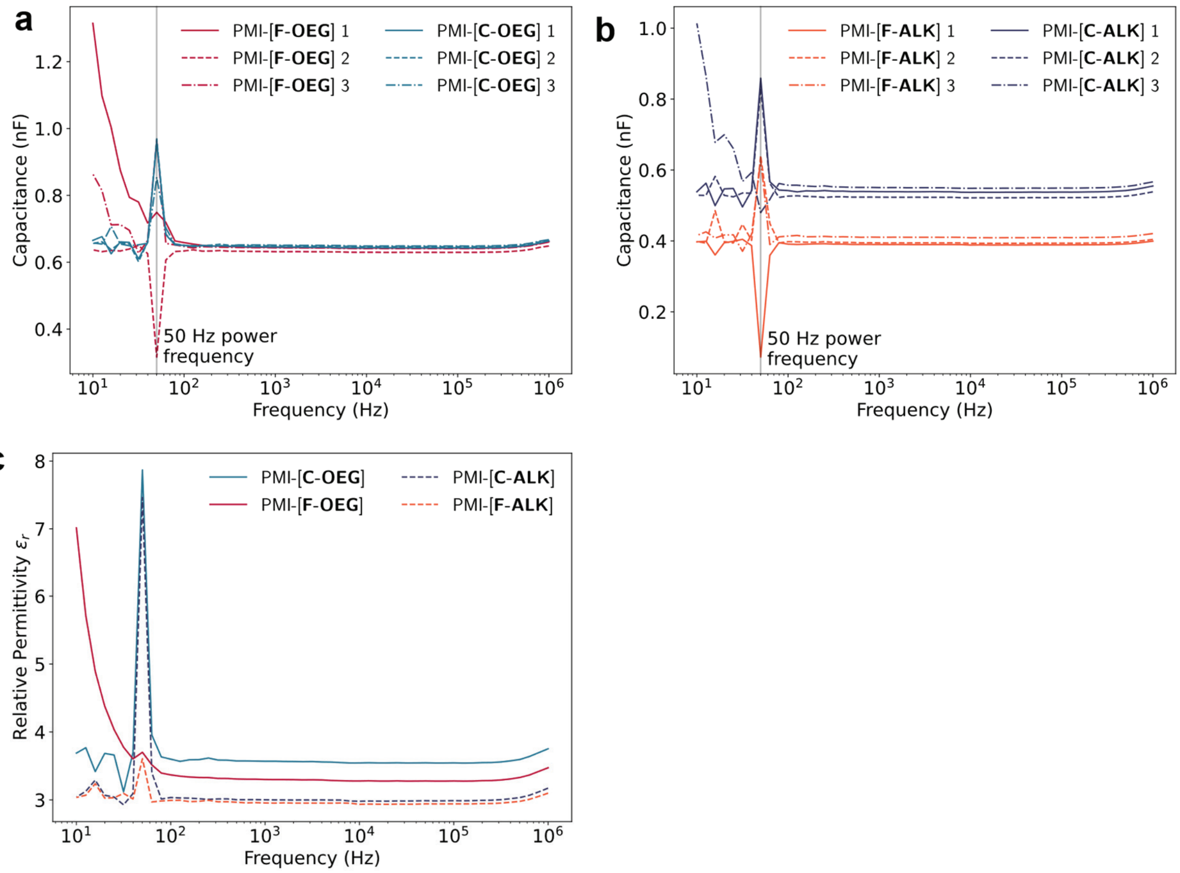

Fig. S20 (**a**) Capacitance of diodes with OEG bearing compounds, (**b**) capacitance of diodes with alkyl chain bearing compounds, (**c**) average relative permittivity for pristine acceptors and reference compounds. The large peak at 50 Hz in all measurements is caused by interference from the AC power supply frequency.

Photovoltaic Properties

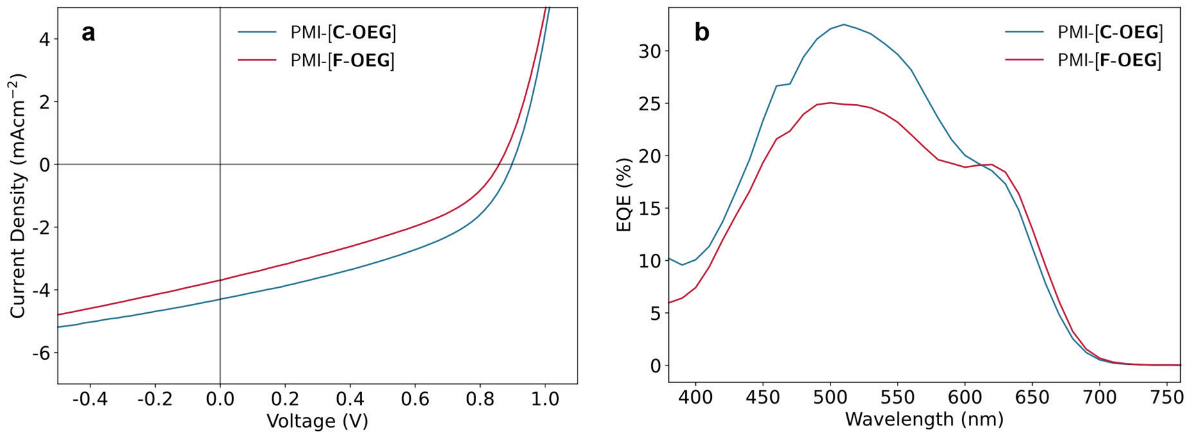

Fig. S21 (**a**) *JV* curves and (**b**) EQE spectra of non-annealed OSCs for both new acceptors. Used was the same architecture as for all other cells (ITO/ZnO/PBDB-T:acceptor/MoO_x/Ag)

References

1. Zhang Y, Chen B, Zhang Y, Qin L, Liu B, Ni B, Gao G (2018) Green Chem 20:1594
2. Liu J, Jiang P, Wang Y, Tu G (2019) Chinese Chem Lett 31:119
3. Schweda B, Reinfelds M, Hofinger J, Bäumel G, Rath T, Kaschnitz P, Fischer RC, Flock M, Amenitsch H, Scharber MC, Trimmel G (2022) Chem Eur J 28:e202200276
4. Weber S, Hofinger J, Rath T, Reinfelds M, Pfeifer D, Borisov SM, Fürk P, Amenitsch H, Scharber MC, Trimmel G (2020) Mater Adv 1:2095
5. Sami S, Alessandri R, Broer R, Havenith RWA (2020) ACS Appl Mater Interfaces 12:17783
